# Genome-wide association study and functional validation of *CsAGD6* conferring drought tolerance in tea plant

**DOI:** 10.1093/hr/uhaf320

**Published:** 2025-11-21

**Authors:** Jiaxuan Yue, Shan He, Shicai Liang, Yu Wang, Huan Wang, Xuxu Lang, Kai Fan, Jianhui Hu, Jiazhi Shen, Litao Sun, Shibo Ding, Zhaotang Ding, Wenjun Qian

**Affiliations:** College of Horticulture, Qingdao Agricultural University, Qingdao 266109, China; College of Horticulture, Qingdao Agricultural University, Qingdao 266109, China; College of Horticulture, Qingdao Agricultural University, Qingdao 266109, China; College of Horticulture, Qingdao Agricultural University, Qingdao 266109, China; College of Horticulture, Qingdao Agricultural University, Qingdao 266109, China; College of Horticulture, Qingdao Agricultural University, Qingdao 266109, China; College of Horticulture, Qingdao Agricultural University, Qingdao 266109, China; College of Horticulture, Qingdao Agricultural University, Qingdao 266109, China; Tea Research Institute, Shandong Academy of Agricultural Sciences, Jinan 250100, China; Tea Research Institute, Shandong Academy of Agricultural Sciences, Jinan 250100, China; Tea Research Institute, Rizhao Academy of Agricultural Sciences, Rizhao 276800, China; College of Horticulture, Qingdao Agricultural University, Qingdao 266109, China; College of Horticulture, Qingdao Agricultural University, Qingdao 266109, China

## Abstract

Drought stress significantly threatens tea production and quality worldwide. To elucidate the genetic basis of drought tolerance in tea plant, we evaluated 11 physiological traits across 115 diverse tea accessions under drought conditions. A comprehensive drought resistance index (*D*-value) was constructed through principal component analysis and fuzzy membership function. Genome-wide association studies identified 67 significant SNPs and pinpointed four candidate genes associated with drought-responsive traits. Integrated transcriptome and qRT-PCR analyses revealed that three genes, including *CsAGD6*, were significantly upregulated under drought stress. Functional assays confirmed that *CsAGD6*, encoding a nucleus-localized ARF-GAP protein, positively regulates drought tolerance by modulating photosynthetic efficiency and membrane stability. Haplotype analysis identified favorable alleles Hap-P1 and Hap-C1 in the promoter and coding regions of *CsAGD6*, respectively. Moreover, an SNP-kompetitive allele-specific PCR marker targeting chr10:206216541 (C/T) was developed and validated in 104 accessions, demonstrating high efficacy for early selection of drought-tolerant genotypes. This study provides novel insights into the molecular mechanisms of drought tolerance in tea plant and offers valuable genetic resources and tools for marker-assisted breeding.

## Introduction

Drought is one of the most critical environmental stresses limiting the growth, yield, and quality of the tea plant (*Camellia sinensis*), particularly under global climate change. Under drought stress, the tea plant exhibits adaptive responses in phenotypic traits and physiological–biochemical characteristics, such as reduced net photosynthesis rate (Pn), transpiration rate (Trmmol), and stomatal conductance (Cond), coupled with elevated malondialdehyde (MDA) levels. These parameters serve as key indicators of drought resistance [1–3]. Drought-tolerant cultivars (varieties) generally maintain higher photosynthetic activity and lower oxidative damage compared to sensitive ones. However, drought resistance in tea plant is a complex quantitative trait governed by multiple genes and interacting factors, and focusing solely on individual physiological parameters may result in biased or incomplete assessments [4, 5]. For more accurate drought resistance evaluation, integrated principal component analysis (PCA)-membership function approaches have been introduced: PCA identifies key traits and membership functions quantify resistance levels. This framework, proven effective in crops like lettuce [6] and sorghum [7], holds promise for tea plant, providing a comprehensive basis for breeding drought-tolerant cultivars.

Recent advances in multiomics technologies have further propelled our understanding of the molecular and genetic mechanisms underlying drought tolerance in tea plant. For example, integrating datasets from the DNA methylome, transcriptome, proteome, and phosphorylated proteome has helped reconstruct multilayer regulatory networks under drought stress [8]. These studies have identified key genes involved in diverse processes, including photosynthesis, hormone signalling, and secondary metabolism. Proteomic analysis further revealed 33 drought-responsive proteins in ABA-pretreated tea leaves, including the upregulation of Rubisco, Hsp70, and lipid-transport proteins, which contribute to enhanced drought tolerance [9]. In addition, CsMOF1 was shown to translocate to the nucleus under drought, where it binds the *CsGS1* promoter and represses its activity, resulting in dynamic changes in theanine biosynthesis and offering a MYB-mediated brake on tea quality under water deficit [10]. Another layer of regulation occurs through glycosylation, where CsUGT87A1-mediated IAA glycosylation and UGT71A59-driven eugenol glucosylation jointly enhance drought resilience by fine-tuning auxin-ABA homeostasis [11]. Similarly, small RNAs are key drought regulators. Wen *et al.* [12] reported that csn-miR156f-2-5p is downregulated by drought, and its knockout impairs Fv/Fm and chlorophyll content, elevates ROS and proline, and targets CsSPL14. These studies illuminate the regulatory networks involved, but forward-genetics strategies are needed to fully resolve the genetic complexity.

In contrast to reverse genetics, GWAS leverage linkage disequilibrium (LD) to associate resequencing-derived polymorphic markers (single nucleotide polymorphisms [SNPs], InDels, CNVs) with phenotypic traits, thereby pinpointing chromosomal regions or loci linked to trait variation. GWAS has been widely applied to dissect quality, yield, and resistance traits in crops like rice [13] and cassava [14]. For example, a GWAS on 362 maize inbred lines identified 40 survival-associated SNPs and 150 candidates, including ZmGRAS15, a drought-responsive GRAS transcription factor verified via transcriptomics to enhance root growth and seedling drought tolerance [15]. Similarly, using EMMAX on 193 *Brassica juncea* accessions [16], identifying 11 drought-responsive genes, including SAUR proteins (BjuA040852, BjuA014514) involved in cell elongation and a LEA protein encoded by BjuB039062.

In tea plant, GWAS application began more recently and has primarily focused on agronomic and quality traits. Combined QTL mapping in an F_1_ population with GWAS in 115 tea accessions to identify a key QTL (qTBF4-1) for bud-burst timing (TBF), narrowing the locus to 188.549–189.369 Mb and nominating CSS00001166 as the most likely candidate gene [17]. Specific site amplification fragment sequencing and GWAS of 200 hybrid offspring from ‘QianMei 601’ identified 12 SNPs and 7 candidate genes, including hormone-responsive ARP and WRKY-ARP [18]. More recently, Zhang *et al.* [19] pinpointed CsKNOX6 as a key suppressor of bud and leaf growth, with overexpression experiments confirming its role in reducing organ size.

For quality traits, studies have shown [20] quantified 2837 metabolites across 215 accessions and identified mQTLs, validating CsUGTa/b and CsCCoAOMT involved in flavonoid biosynthesis. Kong *et al.* [21] analyzed 1325 accessions, correlating copy-number variation of CsTSI with theanine content, and uncovering the CsMYB75-CsTT8 complex regulating catechin and anthocyanin synthesis. In addition, a GWAS of 329 accessions identified 44 SNPs linked to dihydroxy catechins and nominated CsRNF144, an E3 ligase-enhancing photosynthesis and catechin accumulation [22]. Another study of 359 Guizhou accessions highlighted CsAK, a plasmamembrane aspartokinase, as a critical regulator of caffeine biosynthesis [23].

Compared to agronomic and quality traits, phenotyping for stress resistance is more laborious, resulting in fewer GWAS studies on stress traits. In the past two years, studies have conducted GWAS analyses on cold tolerance, identifying LUX ARRHYTHMO (LUX), activated by CsCBF1 and regulating CsLOX2 and jasmonic acid dynamics, to enhance cold resistance [24]. In addition, an analysis of 92 accessions identified 10 cold-associated SNPs and 231 UGT genes. Proteomics confirmed CsUGT71A60, regulated by ARR transcription factors via CRM elements, as key for cytokinin glycosylation and cold tolerance [25]. Although GWAS has advanced our understanding of stress traits in tea plant, there remains a significant gap in drought-resistance studies.

ADP-ribosylation-factors (ARFs) are small GTP-binding proteins within the Ras superfamily, playing critical roles in membrane trafficking, cytoskeletal dynamics, and signalling by cycling between inactive GDP-bound and active GTP-bound states [26]. This switch is tightly regulated by guanine nucleotide exchange factors (GEFs), which facilitate activation, and GTPase-activating proteins (GAPs), which promote GTP hydrolysis and inactivation [27–30]. In their active state, ARFs bind membranes via an N-terminal amphipathic helix, while GAPs induce their detachment by stimulating GTP hydrolysis [26]. In *Arabidopsis thaliana*, 15 AtAGDs proteins have been identified [31, 32]. For instance, the PM-localized AGD1 regulates root hair polarity via its PH domain [33], and AGD12 participates in calcium-activated gravitropic signalling [34]; meanwhile, AGD5 directly regulates ARF1 at the trans-Golgi network [35], and AGD7 promotes ARF1 inactivation to affect COPI vesicle dynamics [26, 28, 32]. AGD6, a class II AGD member, shares conserved GAP domains with AGD7, suggesting functional similarity, though its role has remained unclear [26]. In rice, OsZAC, homologous to AGD12, functions as an ArfGAP protein interacting with GN1.1 and modulating polar auxin transport. OsZAC stabilizes GN1.1, influencing auxin content in young panicles. Knockout of OsZAC increases grain number and panicle size, highlighting its crucial role in regulating rice yield through auxin transport mechanisms [36].

In this study, GWAS of 115 tea accessions based on 11 drought-related traits identified drought-associated SNPs, candidate genes, and beneficial haplotypes. We further designed kompetitive allele-specific PCR (KASP) markers for drought-resistance screening. Our findings establish CsAGD6 as a key positive regulator of drought tolerance, providing both a physiological-genetic framework for tea plant-drought resistance and practical SNP-KASP markers for breeding. Together, these results offer both genetic insights and molecular tools to accelerate the development of drought-resilient tea plant cultivars.

## Results

### Physiological responses to drought stress and evaluation of drought resistance of tea accessions

**Figure 1:**
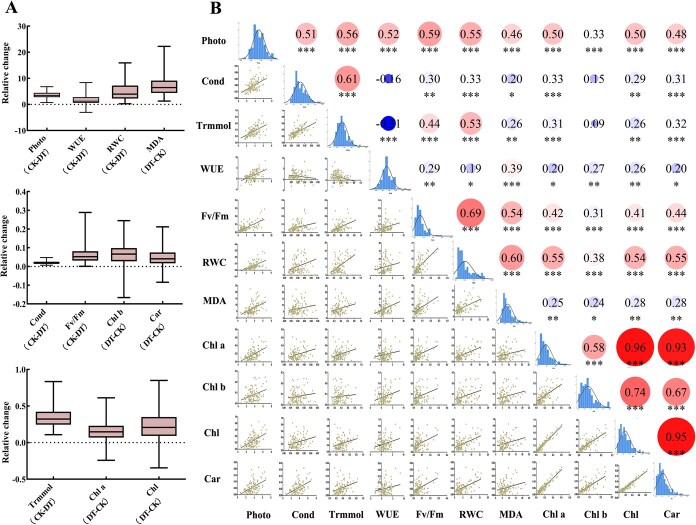
**Analysis of drought resistance-related indicators in tea plant.** (A) Box plots illustrating differences in drought resistance-related indicators among tea accessions; (B) Distribution patterns and correlation analysis of differences in 11 drought resistance-related indicators. The lower-left panels display linear regression statistics between each pair of indicators. The diagonal histograms show the distribution of differences. The upper-right panels present the correlation coefficients, with asterisks denoting levels of statistical significance (^*^P < 0.01, ^**^P < 0.005, ^***^P < 0.001).

To investigate the physiological responses of the tea plant to drought stress, 115 tea accessions were subjected to drought treatment, and changes in various physiological indicators were compared before and after the treatment. The results showed that after drought stress, Pn, Trmmol, Cond, relative water content of leaves (RWC), and Fv/Fm decreased, whereas MDA content significantly increased, indicating that drought inhibits photosynthesis in tea plant and may lead to cell membrane damage and intensified oxidative stress. Furthermore, chlorophyll a (Chl a), chlorophyll b (Chl b), total chlorophyll (Chl), and carotenoid (Car) content increased in most tea accessions, though a few showed decreased pigment levels, indicating variation in physiological responses among different germplasm under drought stress. The trend of WUE variation differed among accessions (Fig. 1A).

PCA reduced the variation in 11 standardized indicators to three principal components, accounting for a cumulative contribution of 78.417%. The first principal component explained 48.432% of the total variance and was mainly associated with Pn, Fv/Fm, RWC, Chl a, Chl b, total Chl, and Car, highlighting their importance in evaluating drought resistance. Among these, the chlorophyll index had the highest absolute loading value. The second principal component accounted for 16.194% of the variance and was primarily associated with Cond and Trmmol. The third principal component explained 13.792% of the variance and was mainly related to WUE and MDA (Table S1). By calculating the comprehensive evaluation *D*-value for each tea plant germplasm (Table S2), it was found that higher *D*-values corresponded to stronger drought resistance, providing a basis for selecting drought-tolerant germplasm.

Correlation analysis revealed significant associations among most drought resistance traits, except for Cond with WUE and Chl b, and Trmmol with Chl b. Pn showed significant positive correlations with most indicators, and a strong correlation (*r* = 0.96) was observed among the four photosynthetic pigment indicators, indicating a close relationship in physiological responses during drought stress. Additionally, MDA exhibited significant or highly significant positive correlations with all physiological traits except Cond and Trmmol, suggesting that membrane lipid peroxidation plays a crucial role in the drought response of the tea plant. Moreover, the distribution of differences in physiological traits approximated a normal distribution (Fig. 1B), indicating suitability for GWAS and providing a robust data foundation for subsequent gene mining related to drought resistance.

### Genetic structure analysis

To facilitate the GWAS of drought resistance traits in tea plant, a combined set of whole-genome resequencing data from 115 diverse tea accessions was analyzed. The sequencing data were aligned to the reference genome (‘Lucha 6’), resulting in the identification of 51 458 813 SNPs. After quality filtering, 28 383 000 high-confidence SNPs were retained for downstream analyses.

To elucidate the genetic relationships among the tea accessions, a phylogenetic tree and PCA were employed. Both analyses consistently grouped the 115 tea accessions into four distinct subpopulations, which corresponded closely to their geographic origins. Subgroup I consisted of 17 accessions, with 11 originating from Zhejiang province. Subgroup II comprised 57 accessions, predominantly from Shandong province. Subgroup III included 35 accessions, also mainly from Zhejiang province. Subgroup IV contained 6 accessions distributed across Zhejiang, Anhui, and Shandong provinces.

Population structure analysis, performed using Bayesian clustering, supported the classification of the accessions into four subgroups, with *K* = 4 identified as the optimal number of clusters based on cross-validation error. Except for one branch comprising 13 accessions originally grouped under subgroup II in the phylogenetic analysis but reassigned to subgroup I in the population structure analysis, the classifications were generally consistent across methods. This concordance underscores the robustness of the subgroup classification and highlights the genetic differentiation shaped by geographic distribution.

**Figure 2:**
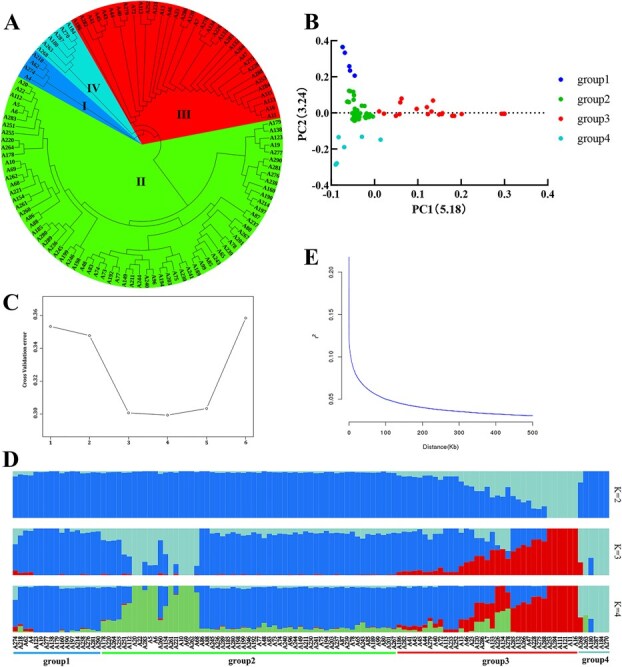
**Genetic structure analysis of tea accessions.** (A) Phylogenetic tree of 115 tea accessions; (B) PCA based on SNP data; (C) Cross-validation error plot for determining the optimal number of subgroups (K); (D) Population structure analysis based on Bayesian clustering; and (E) LD decay curve for the population.

LD analysis was further conducted to evaluate the genetic architecture of the population. The analysis revealed that the maximum average LD coefficient (*r*^2^) across the population was 0.218. As *r*^2^ declined to 0.109, the corresponding physical distance was approximately 1100 bp, indicating the extent of LD decay within the germplasm population (Fig. 2E). This LD pattern provides a reference for determining the mapping resolution in subsequent GWAS.

### GWAS analysis

To identify genetic loci associated with drought resistance traits in tea plant, GWAS was conducted using the linear mixed model (LMM) implemented in GEMMA. The analysis included phenotypic data for 11 drought resistance-related traits and *D*-value, and genotypic data from 28 383 000 high-quality SNPs across 115 tea accessions. Bonferroni correction was applied to control for multiple testing, with a genome-wide significance threshold set at *P* < 1.76 × 10^−9^ (−log₁₀*P* = 8.75) and a suggestive threshold at *P* < 3.52 × 10^−9^ (−log₁₀*P* = 8.45), the latter of which is marked by a red line in the Manhattan plots.

**Figure 3:**
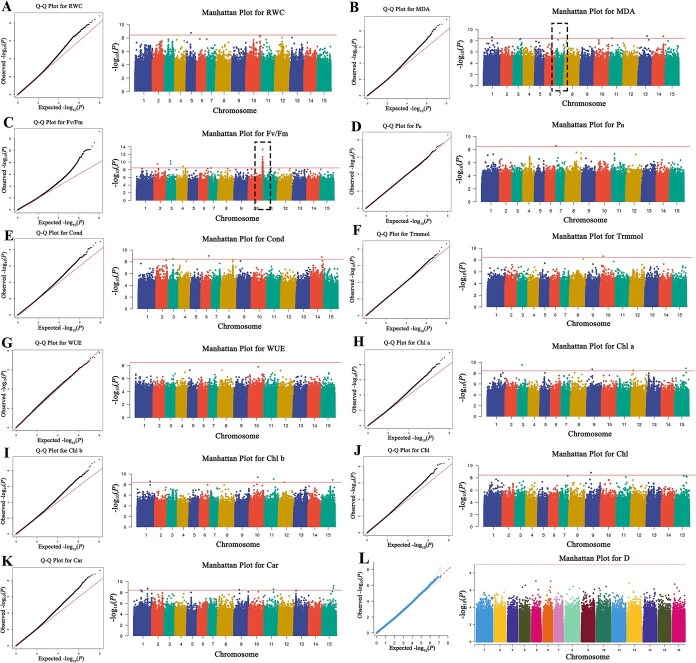
**Genome-wide association study of drought resistance-related traits in tea plant.** (A–K) Quantile-quantile (Q-Q) plots and Manhattan plots for 11 drought resistance-related traits; (L) Manhattan plot summarizing all associated SNPs. The suggestive association threshold is indicated by dashed horizontal lines in the Manhattan plots. SNP hotspots are annotated on relevant chromosomes.

GWAS identified 79 SNPs associated with drought resistance traits, 67 of which exceeded the genome-wide threshold (Fig. 3 and Table S3). Manhattan plots illustrated the distribution of these associations across chromosomes, while quantile–quantile (Q-Q) plots showed that the majority of SNPs followed the expected null distribution, indicating robust control of type I error and minimal genomic inflation. Only a limited number of SNPs exhibited upward deviation from the diagonal line, suggesting strong true-positive signals. Among the associated loci, several SNPs showed multitrait associations, suggesting potential pleiotropic effects. For example, SNP chr15:157502868 was simultaneously associated with Chl a and Car content, while SNP chr11:130671257 was linked to Chl b and Car content. Additionally, SNP chr9:76677440 showed associations with Chl a, Chl, and Car levels, indicating its possible involvement in coordinated regulation of pigment metabolism under drought stress. Furthermore, a notable QTL hotspot was detected at the distal end of chromosome 10 (Fig. 3C), highlighting a key genomic region that may contribute significantly to drought tolerance.

### Screening and expression analysis of candidate genes related to drought resistance

Based on the GWAS results, genomic regions spanning 50 kb upstream and downstream of each significant SNP were defined as candidate intervals for further gene mining. Within these regions, a total of four key candidate genes were identified near 67 significantly associated loci (Fig. S1A). Two of these genes (augustus080390 and augustus080391) located on chromosome 10 were significantly associated with chlorophyll fluorescence-related traits. These genes were annotated as *CsNHX1*, encoding a cation/H^+^ antiporter, and *CsAGD6*, encoding an ADP-ribosylation factor GTPase-activating protein. Additionally, two candidate genes (augustus090625 and augustus090626) located on chromosome 7, were significantly associated with MDA content and annotated as disease resistance proteins *CsRPP13*-*LK3* and *CsRPP13*-*LK4*, respectively (Table S4). Promoter analysis revealed the presence of multiple drought-responsive cis-regulatory elements in the upstream regions of all four genes, suggesting their involvement in the drought stress response of the tea plant (Table S5 and Fig. S1B).

**Figure 4:**
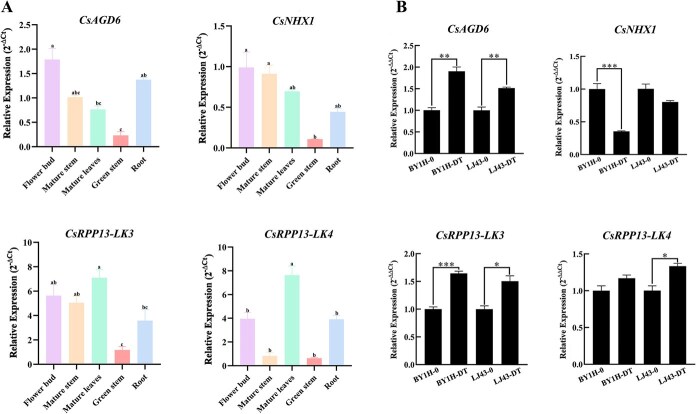
**Expression profiling of candidate drought-responsive genes in tea plant.** (A) Tissue-specific expression patterns of four candidate genes across various organs of the tea plant; (B) Expression levels of candidate genes under drought stress conditions. Asterisks indicate significant differences compared to the control (^*^P < 0.05; ^**^P < 0.01). ‘0’ represents before drought stress and ‘DT’ represents after drought stress. ‘BY1H’is ‘Baiye 1’and ‘LJ43’ is ‘Longjing 43′.

Tissue-specific expression profiling further illustrated the expression characteristics of these candidate genes across different tissues of the tea plant (Fig. 4A). All four genes were expressed in various tissues, with overall low expression levels in green stems. Notably, *CsAGD6* showed relatively high expression in flower buds and roots, indicating its potential roles in floral development and root-specific signalling or metabolism. *CsNHX1* exhibited higher expression in flower buds and mature stems, suggesting involvement in ion homeostasis and signalling during tissue maturation. *CsRPP13*-*LK3* and *CsRPP13*-*LK4* shared broadly similar expression patterns; however, *CsRPP13*-*LK3* had distinctly higher expression in mature stems compared to *CsRPP13*-*LK4*, implying possible functional divergence between these homologous genes.

To explore their responsiveness to drought stress, drought treatment experiments were conducted using the drought-tolerant cultivar ‘Longjing 43’ and the drought-sensitive cultivar ‘Baiye 1’. Gene expression levels were assessed by RNA-seq and further validated by qRT-PCR analysis, with qRT-PCR results supporting the reliability of the transcriptome data (Fig. S2). The results revealed divergent transcriptional responses to drought: the expression of *CsNHX1* was significantly downregulated after drought stress, especially in ‘Baiye 1’, suggesting that its suppression might be linked to higher drought sensitivity. In contrast, *CsAGD6*, *CsRPP13*-*LK3*, and *CsRPP13*-*LK4* were all markedly upregulated under drought conditions in both cultivars, highlighting their potential roles in enhancing drought tolerance (Fig. 4B).

### Functional characterization of *CsAGD6* under drought stress

**Figure 5:**
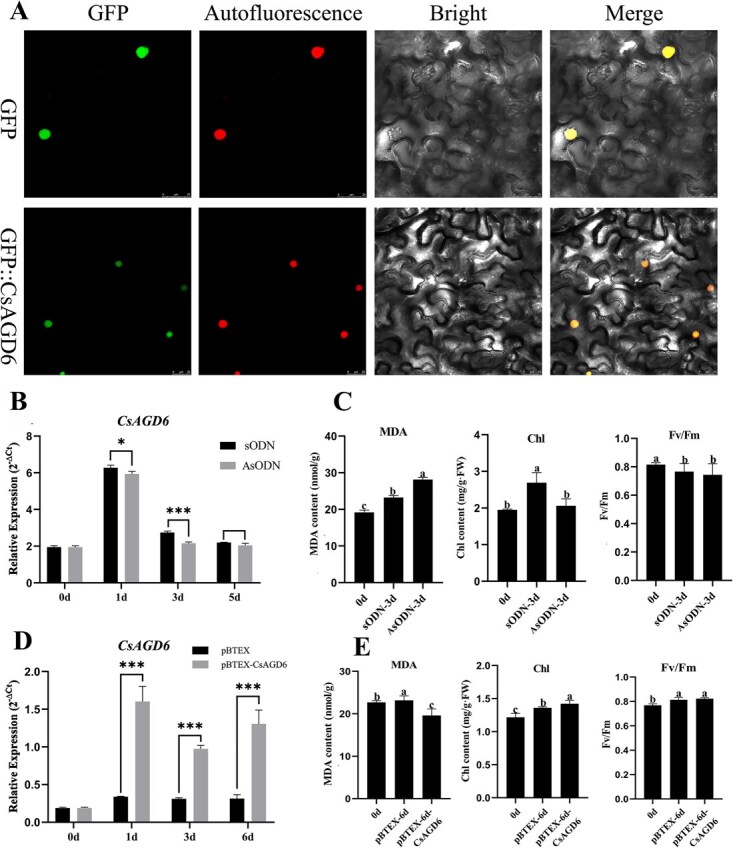
**Subcellular localization and transient functional validation of *CsAGD6* under drought stress in tea plant.** (A) Subcellular localization of CsAGD6-GFP fusion protein in tobacco epidermal cells; (B) Relative expression level of *CsAGD6* after transient gene silencing using antisense oligonucleotides (AsODN); (C) Physiological measurements in *CsAGD6*-silenced tea leaves after drought stress; (D) Relative expression level of *CsAGD6* in tea leaves transiently overexpressing *CsAGD6*; (E) Drought-related physiological indices in *CsAGD6*-overexpressing plants. Asterisks and different letters indicate significant differences at *P* < 0.05.

To investigate the functional role of *CsAGD6* in drought resistance, subcellular localization and transient expression analyses were performed. The subcellular localization of *CsAGD6* was determined in tobacco epidermal cells (*Nicotiana benthamiana*) via transient expression assays. Figure 5A shows that *CsAGD6* is predominantly localized on the cell nucleus in *Nicotiana benthamiana*, consistent with the prediction results presented in Table S5.

Functional validation through transient gene silencing showed that, after 3 days of simulated drought stress, the expression level of *CsAGD6* in the antisense oligonucleotide (AsODN) treatment group was significantly reduced compared to the sense control (sODN), confirming effective gene silencing both in tea leaves of ‘Baiye 1’ and ‘Longjing 43’ (Figs 5B and S5). Physiological measurements revealed that MDA content significantly increased in the AsODN-treated plants, whereas both Chl and Fv/Fm were markedly reduced (Figs 5C and S5), suggesting that silencing *CsAGD6* compromises drought tolerance by aggravating oxidative damage and impairing photosynthetic performance.

To further verify the regulatory role of *CsAGD6*, transient overexpression experiments were conducted both in ‘Baiye 1’ and ‘Longjing 43’. The results showed that *CsAGD6* expression was significantly elevated in overexpressed plants compared to the empty vector control (Figs 5D and S5). Correspondingly, overexpression led to a significant decrease in MDA content and higher Chl and Fv/Fm levels under drought stress (Figs 5E and S5), indicating enhanced membrane stability and photosynthetic efficiency. Collectively, these findings demonstrate that *CsAGD6* positively regulates drought tolerance in tea plant, likely through its nuclear function in modulating cellular stress responses and protecting photosynthetic machinery.

### Haplotype analysis of *CsAGD6* reveals allelic variation related to drought tolerance

**Figure 6:**
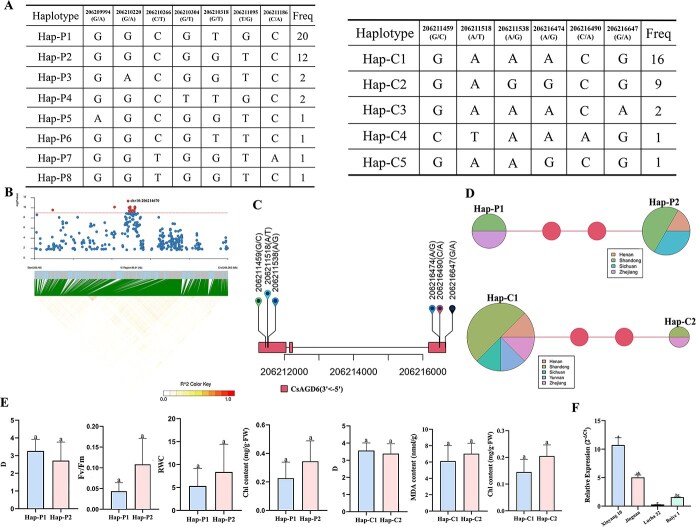
**Haplotype analysis of *CsAGD6* and its association with drought resistance in tea germplasms.** (A) Haplotype structure and frequency of *CsAGD6* based on SNPs in the promoter and coding regions; (B) Regional LD block analysis; (C) Genomic structure of *CsAGD6* and distribution of major SNP loci; (D) Haplotype network diagrams and frequency pie charts showing the distribution of promoter (Hap-P) and coding region (Hap-C) haplotypes; (E) Phenotypic comparison of major haplotypes under drought stress; (F) Relative expression levels of *CsAGD6* in representative tea accessions from Hap-C1 and Hap-C2. Different letters indicate significant differences at *P* < 0.05.

To further elucidate the genetic basis underlying *CsAGD6*-mediated drought resistance, haplotype analysis was conducted based on 287 tea accessions using resequencing data. LD analysis showed that *CsAGD6* was located within a strong LD block (206.165–206.265 Mb), consistent with the association peak identified in the GWAS (Figs 3C and 6B). In total, 16 SNPs in the promoter region and 15 non-synonymous mutations in the coding region of *CsAGD6* were detected, which formed eight promoter haplotypes (Hap-P1 to Hap-P8) and five coding region haplotypes (Hap-C1 to Hap-C5) (Table S6). Among them, Hap-P1, Hap-P2, Hap-C1, and Hap-C2 were identified as the major haplotypes based on their frequencies (Fig. 6A).

Haplotype network analysis revealed that genetic variation in *CsAGD6* exhibited obvious geographical differentiation. Specifically, Hap-P1 and Hap-C2 were predominantly distributed in two provinces, Hap-P2 in three provinces, and Hap-C1 in five provinces (Figs 6D and S6). This regional distribution likely reflects ecological adaptation and selection pressure on *CsAGD6* variants in different environments.

Phenotypic association analysis showed that germplasm carrying Hap-P2 exhibited greater declines in Fv/Fm (0.102 vs. 0.044), RWC (8.367% vs. 5.322%), and Chl (0.378 vs. 0.226) after drought treatment compared to Hap-P1, indicating more severe photosynthetic impairment. Furthermore, the comprehensive drought tolerance index (*D*-value) was higher in Hap-P1 (3.265) than Hap-P2 (2.719) (Fig. 6E). Similarly, Hap-C1 performed better than Hap-C2 in terms of lower changes in MDA (6.488 vs. 7.012), Chl (0.143 vs. 0.205), and higher *D*-value (3.567 vs. 3.385), suggesting superior drought resistance in germplasms carrying Hap-C1.

Moreover, qRT-PCR analysis showed that *CsAGD6* expression levels in Hap-C1 genotypes (e.g. ‘Xinyang 10’ and ‘Jinguan’) were significantly higher than those in Hap-C2 genotypes (e.g. ‘Lucha 32’ and ‘Baiye 1’), consistent with the phenotypic performance (Fig. 6F). These results support that Hap-P1 and Hap-C1 represent favorable haplotypes of *CsAGD6* for drought tolerance. Although haplotype combination analysis (Table S7) indicated no clear epistatic interaction among haplotype pairs, this aspect warrants further investigation with larger phenotypic datasets.

### Development and validation of SNP-KASP marker for *CsAGD6* drought-responsive allele

**Figure 7:**
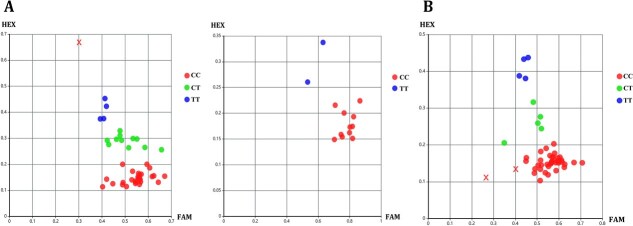
**Genotyping and application of *CsAGD6*-derived SNP-KASP marker in tea accessions.** (A) Genotyping of 53 tea accessions using the SNP-KASP marker; (B) Application of the chr10:206216541 marker to genotype 51 elite tea accessions.

To facilitate the molecular identification of drought-tolerant alleles in tea plant, a total of 79 candidate SNP loci were extracted from the reference genome and converted into KASP markers according to standardized primer design criteria. Among them, the SNP marker targeting chr10:206216541 (C/T) (Table S8), located in the exon region of *CsAGD6*, exhibited excellent genotyping resolution across 53 tea germplasm accessions with known drought resistance. This marker effectively distinguished three genotypes: CC, CT, and TT, with fluorescence signal clusters corresponding to each genotype clearly separated in the scatter plot. All accessions, except ‘Mingshanzao 311’, produced clear signals (Fig. 7A). Notably, germplasms with stronger drought resistance were predominantly of the CC genotype, while those with weaker drought resistance carried CT or TT genotypes (Table S9), indicating a significant association between genotype and drought tolerance.

To evaluate the marker’s applicability in broader germplasm screening, the chr10:206216541 marker was applied to 51 elite tea accessions maintained in our collection. As shown in Fig. 7B, 40 samples carried the CC genotype, 5 were CT, 4 were TT, and 2 samples failed to amplify. These results demonstrate that the chr10:206216541 (C/T) marker can serve as a reliable tool for preliminary screening of drought-tolerant germplasms, thereby providing a foundation for marker-assisted selection (MAS) in tea breeding programs.

## Discussion

### Comprehensive evaluation of drought resistance in tea plant based on the membership function method

Drought stress represents one of the major abiotic factors limiting the growth and productivity of the tea plant. In the present study, we systematically evaluated 11 physiological indicators across 115 tea accessions under drought stress and developed a *D*-value evaluation system based on PCA and the membership function method. This integrated approach provides a robust model for quantifying drought resistance and offers a reliable basis for identifying drought-tolerant germplasms [37, 38].

Under drought stress, physiological traits such as Pn, Cond, Trmmol, RWC, and Fv/Fm significantly decreased. These results are consistent with previous findings that drought reduces photosynthesis by inhibiting CO₂ assimilation, inducing stomatal closure, and damaging photosynthetic structures [3, 6, 39]. Furthermore, a notable accumulation of MDA was observed, indicating the occurrence of oxidative stress and membrane lipid peroxidation, which are key consequences of drought-induced physiological damage in tea plant [40]. Interestingly, Chl a, Chl b, Chl, and Car increased in most germplasm resources, suggesting that certain tea germplasms may adapt to drought stress by activating pigment-mediated photoprotective mechanisms [41]. Analysis of variation coefficients revealed a wide range across the 11 traits (31.7% to 79.9%; Table S1), reflecting substantial phenotypic diversity among the germplasms. Notably, Fv/Fm exhibited the highest coefficient of variation (79.9%), indicating it may be particularly sensitive to multigenic regulation or environmental influence.

PCA effectively reduced the dimensionality of the 11 traits into three principal components, cumulatively explaining 78.417% of the total variation. The first principal component (48.432%) was strongly associated with photosynthetic and pigment-related traits (Pn, Fv/Fm, RWC, Chl a, Chl b, total Chl, and Car), highlighting their central role in drought resistance evaluation. The second principal component (16.194%) captured traits related to stomatal regulation (Cond, Trmmol), while the third component (13.792%) reflected indices associated with water use efficiency (WUE) and oxidative stress (MDA). Correlation analysis revealed that Pn was significantly and positively correlated with most other traits, and there was a high degree of synergy among pigment indicators (*r* = 0.96). MDA was significantly correlated with all traits except Cond and Trmmol, further supporting the widespread impact of oxidative stress on physiological responses. Given the interdependence and partial independence among these indicators, a single trait is insufficient to accurately assess drought resistance. Therefore, the integrated *D*-value, derived from PCA and the membership function method, was adopted for comprehensive evaluation. Results showed that higher *D*-values correspond to stronger drought resistance in tea plant. Moreover, *D*-values were strongly correlated with the rankings of photosynthetic D-values (*r* = 0.838), pigment *D*-values (*r* = 0.775), and RWC differences (*r* = 0.761), underscoring the major contributions of these traits to drought-resistance evaluation.

### GWAS analysis reveals genetic basis of drought resistance in tea plant

As a complex quantitative trait, drought resistance is controlled by multiple genes. As a key forward genetics approach, GWAS have been extensively applied to dissect the genetic architecture of complex traits and identify candidate genes conferring drought tolerance in plants. In this study, we performed whole-genome resequencing at 10× coverage depth, resulting in approximately 28 million high-quality SNPs suitable for GWAS. This ensured both the sensitivity and accuracy of SNP detection, minimizing false negatives caused by insufficient sequencing depth. To improve the robustness of association signals, an LMM was used to correct for population structure and kinship, along with false discovery rate correction for multiple testing. Additionally, the genetic correlation and LD patterns of the population were analyzed, as these are crucial for minimizing false-positive associations [42]. Population structure analysis grouped the 115 tea accessions into four genetically distinct subpopulations consistent with their geographical origins, indicating clear genetic stratification and high diversity-favorable conditions for association mapping [43].

LD decay patterns revealed insights into the evolutionary processes shaping the population. In general, a longer LD decay distance indicates slower recombination and stronger selection, whereas shorter distances are associated with more frequent recombination events and weaker selection. Cross-pollinating species typically exhibit faster LD decay than self-pollinating ones, and wild populations decay faster than domesticated ones [44]. In this study, the LD decay distance was approximately 1100 bp, which is shorter than in cultivated tea plants in Guizhou (2 kb) [45] and ancient tea plants (19.3 kb) [46], but longer than in Biluochun tea populations from Dongting Mountain (500 bp) [47]. These results suggest that the tea germplasm in this study has undergone relatively low selection pressure, making it suitable for high-resolution GWAS [48]. By applying Bonferroni correction, a total of 79 SNPs significantly associated with drought resistance were identified, including 67 highly significant loci. Furthermore, four candidate genes were revealed, among which CsAGD6, encoding an ADP-ribosylation factor (ARF) GTPase-activating protein, exhibited strong regulatory effects on drought response.

### Functional characterization of CsAGD6 in tea plant drought response

In plants, ADP-ribosylation-factor GTPase-activating proteins (ARF-GAPs) terminate ARF-GTP signalling by catalyzing GTP hydrolysis, thereby governing vesicle formation, membrane identity and polar trafficking [26]. The Arabidopsis genome encodes fifteen ARF-GAPs (AGD1-15) that fall into seven phylogenetic classes. Functional insights into class-I relatives are more advanced: AtAGD1 localizes to the apical plasma membrane (PM) of root hairs via its pleckstrin-homology domain, where it constrains tip growth by antagonizing ARF activity; loss of AGD1 results in aberrant root hair morphology [33]. Likewise, VAN3/AGD3 localizes to both the PM and the trans-Golgi network/early endosome (TGN/EE), where it antagonizes the ARF-GEF GNOM to regulate vein continuity by polarizing PIN auxin efflux carriers [49]. These examples highlight how ARF-GAPs achieve functional divergence through distinct subcellular localizations and selective ARF recognition. Notably, functional studies of AGD6 orthologues in model plants such as Arabidopsis and rice have primarily implicated them in growth and developmental processes, such as vesicle trafficking and cell polarity maintenance [26,27,49]. For instance, the closest paralogue of AtAGD6, AtAGD7, inactivates ARF1 at the Golgi and is essential for COPI-mediated retrograde transport and Golgi integrity [28]. In contrast, the biological function of AGD6 itself has remained largely elusive, and no AGD6-class protein has previously been linked to abiotic stress adaptation.

In this study, we identified 17 CsAGD genes in the tea plant genome (Fig. S3B and Table S10), all encoding proteins with conserved GAP domains (Fig. S3A), including a highly conserved arginine residue critical for GTP hydrolysis (Fig. S3C) [35]. Phylogenetic analysis clustered *CsAGD6* within class II, adjacent to AtAGD6 and the Glo3p-type clade, confirming its identity as the tea orthologue of AtAGD6. Our functional assays now reveal a novel role for an AGD6-class protein in drought tolerance: silencing *CsAGD6* significantly increased MDA accumulation and reduced photosynthetic efficiency (Chl, Fv/Fm), indicating that its GAP activity is important for maintaining membrane integrity and protecting photosynthetic machinery under water deficit. We propose that *CsAGD6* contributes to drought resilience by modulating ARF-dependent trafficking processes, potentially involving stress-responsive proteins such as aquaporins, ion channels, or ABA signalling components. Alternatively, it may influence cytoskeletal organisation, which is essential for sustaining cell function during osmotic stress. While these mechanisms remain to be elucidated, our data provide a functional entry point for exploring AGD6-mediated stress adaptation. Heterologous expression in *Arabidopsis thaliana* will also be important to validate these mechanisms in vivo. Future work will focus on identifying specific ARF-GTPase substrates of *CsAGD6* and its potential interacting partners through yeast two-hybrid screening and co-immunoprecipitation assays.

Haplotype analysis further revealed two major allelic forms of *CsAGD6* (Hap-P1 and Hap-P2) associated with contrasting drought tolerance. Hap-P1 accessions displayed higher gene expression and stronger drought resistance. In the promoter region, two SNPs (206 210 318 G/T and 206 211 095 T/G) were located within predicted MYB and BBR-BPC transcription factor binding sites (Fig. S4), suggesting *cis*-regulatory variation in *CsAGD6* transcription. Furthermore, we identified two nonsynonymous SNPs in the coding sequence (206 211 538 and 206 216 474). These cause Thr93Ala and His397Arg amino acid substitutions within conserved functional domains, which may affect GAP activity or substrate specificity and thereby fine-tune downstream stress responses. These hypotheses require further validation, for instance via *in vitro* GAP assays, identification of CsAGD6-interacting ARFs or partners, and heterologous expression in *Arabidopsis*.

Taken together, our results provide the first evidence that an AGD6-class ARF-GAP contributes to abiotic stress tolerance, demonstrating that *CsAGD6* safeguards membrane stability and photosynthesis under drought stress. Moreover, the discovery of functional haplotypes highlights how natural allelic variation modulates *CsAGD6* activity and contributes to adaptive drought responses in tea plant. This work not only extends the functional repertoire of ARF-GAPs beyond vesicle trafficking and development but also offers a promising genetic target for improving stress resilience in perennial crops.

### Development and application of a *CsAGD6*-based KASP marker

Given the extended breeding cycle and low efficiency of conventional methods in perennial crops like the tea plant, MAS is essential for accelerating trait improvement. Although various markers have been developed for quality traits, such as ‘CafLess-TCS1’ for low caffeine [50], CAPS for high caffeine content [51], InDel markers for high CI [52], and GWAS-based ‘TBF-dCAPS’ for bud timing [53], molecular markers specific to drought resistance in tea plant remain scarce.

In this study, a KASP marker was developed based on a synonymous SNP (Chr10:206216541, C/T) in the exon of *CsAGD6*. While the mutation does not alter the amino acid sequence, it successfully distinguishes drought-resistant from sensitive germplasms, in line with modern breeding principles favouring linkage strength [54]. The marker achieved 98% accuracy in 53 validation samples and genotyped 96% of a 51-sample germplasm panel, among which 78% of superior drought-resistant accessions carried the CC allele. Such performance enables efficient prescreening without labour-intensive phenotyping. Similar KASP-based strategies have been widely adopted in drought-resistance breeding of wheat [55], pepper [56], and grapevine [57]. The *CsAGD6*-derived KASP marker developed here not only fills a critical gap in stress-resistance breeding tools for the tea plant but also represents a pivotal advancement in implementing MAS strategies for this economically important crop. Nevertheless, to ensure its broad applicability and robustness, further validation in larger and independent populations will be essential, which will strengthen confidence in its stability across diverse genetic backgrounds and breeding environments.

### Conclusion

This study comprehensively evaluated 11 drought-related physiological traits across 115 tea accessions and conducted GWAS based on both individual traits and a composite drought resistance index (*D*-value), identifying 79 significant SNP loci and 4 candidate drought-responsive genes. Transcriptome and qRT-PCR analyses showed that three of these genes, excluding *CsNHX1*, were consistently upregulated under drought stress. Among them, *CsAGD6* was functionally validated as a drought-inducible gene through transient assays. Haplotype analysis revealed that *CsAGD6* haplotypes Hap-P1 and Hap-C1 were associated with superior drought tolerance. Furthermore, an SNP-KASP marker targeting the chr10:206216541 (C/T) locus in *CsAGD6* was successfully developed and shown to reliably distinguish drought-tolerant genotypes across diverse tea accessions. These findings provide new insights into the genetic basis of drought resistance in tea plant and offer practical tools for molecular-assisted breeding.

## Materials and methods

### Plant materials

A total of 115 one-year-old cuttings from different tea plant cultivars (varieties) were used as experimental materials. Detailed information on the cultivars (varieties) is provided in Table S11. All cuttings were cultivated under controlled conditions in the artificial climate chamber at Qingdao Agricultural University. The environmental conditions were maintained at 24°C, with a 16 h light/8 h dark photoperiod and 65% relative humidity.

### Drought stress treatment

Tea seedlings with uniform growth and similar size were selected for natural drought treatment. Prior to drought induction, all seedlings were thoroughly irrigated until water drained from the bottom of the pots. At this point, the soil moisture content was approximately 40%, which was defined as the well-watered control level (Day 0). Two days later, photosynthetic parameters and Fv/Fm were measured on the third to fifth mature leaves below the apical bud. Leaf samples were immediately collected from the same position and snap-frozen in liquid nitrogen for downstream analyses. Subsequently, drought stress was imposed by completely withholding water for 20 consecutive days, while maintaining all other environmental conditions constant. The drought treatment was concluded when the soil moisture content reached approximately 15%, at which point postdrought measurements and sampling were performed on the same plants. Each tea plant cultivar/variety included three biological replicates, with each replicate comprising 15 individual seedlings.

### Determination of photosynthesis parameters, Fv/Fm and MDA content

Photosynthetic parameters were measured on the third to fifth mature leaves using a portable photosynthesis system (Li-6400XT, LI-COR, USA) [58]. Parameters included Pn (μmol CO₂ m^−2^·s^−1^), Trmmol (mmol H₂O m^−2^·s^−1^), and Cond (mol H₂O m^−2^·s^−1^). Water use efficiency (WUE) was calculated as WUE = Pn/Trmmol. The maximum quantum yield of Fv/Fm was determined using a FluorPen FP 110 handheld chlorophyll fluorometer (Photon Systems Instruments, Czech Republic). Prior to measurement, tea plant leaves were dark-adapted for 20 min using light-blocking clips.

MDA content was determined following the method described [59]. Briefly, 0.1 g of fresh leaf tissue was weighed and placed into a 2-ml centrifuge tube with 1 ml extraction buffer. After vortexing and centrifugation, 0.1 ml of the supernatant was mixed with 0.3 ml of Reagent I and incubated in a 95°C water bath for 30 min. After cooling and centrifugation, absorbance of the supernatant was measured at 532 and 600 nm, recorded as A_532_ and A_600_, respectively. MDA concentration was calculated using the following formula:


\begin{align*} \textrm{MDA (nmol/g)}=51.6\times{\textrm (}A_{532}-A_{600}{\textrm )}/W, \end{align*}

where *W* represents the fresh weight of the sample (g). Each cultivar/variety was tested in triplicate, with each replicate consisting of 15 seedlings.

### RWC and photosynthetic pigment content

Prior to and following the drought stress treatment, the third to fifth mature leaves below the apical bud were collected from each tea plant cultivar/variety to determine RWC, as well as Chl and Car concentrations.

RWC is carried out in accordance with the described method [60]. Fresh leaves were collected and their fresh weight (*W*₀) was measured immediately to a precision of 0.1 g. The samples were then oven-dried at a constant temperature until a stable dry weight (*Wd*) was achieved. After cooling to room temperature in a desiccator, *Wd* was recorded. RWC was calculated using the formula:


\begin{align*} \textrm{RWC (\%)}=(W_0 - Wd)/W_0 \times 100. \end{align*}

Chl and Car contents were determined using the 95% ethanol extraction method. Approximately 0.1 g of fresh leaf tissue was weighed and incubated in 10 ml of 95% ethanol in the dark for 17 h until complete decolorization of the leaves. The absorbance of the extract was then measured at 470, 649, and 665 nm, recorded as *A*_470_, *A*_649_, and *A*_665_, respectively, using 95% ethanol as a blank control.

The pigment concentrations were calculated using the following equations:

Chl a content (Ca, mg/L) = 13.95 × *A*_665_ − 6.88 × *A*_649_.

Chl b content (Cb, mg/L) = 24.96 × *A*_649_ − 7.32 × *A*_665_.

Car content (Cc, mg/L) = [1000 × *A*_470_ − 2.05 × Ca − 114.8 × Cb] / 245.

The final pigment contents were expressed in mg/g fresh weight and calculated as: 


\begin{align*} \textrm{Chl content (mg/g}\!\cdot\! FW=(C\times V)/(W \times 1000) \end{align*}

where *C* represents the pigment concentration (mg/L), *V* is the volume of extract (ml), and *W* is the fresh weight of the sample (g). Each tea plant cultivar/variety included three biological replicates, with each replicate comprising 15 seedlings.

### Evaluation of drought resistance

Drought resistance of each tea plant germplasm resource was evaluated based on a comprehensive *D*-value calculated from 11 physiological indicators, following the method described [40]. To eliminate unit differences among traits, all indicators were first normalized using the following equation:

[1] *Y_ij_* = (*X*_*j*max_ − *X_ij_*) / (*X*_*j*max_ − *X*_*j*min_).

where *X_ij_* represents the value of the *j*-th indicator for the *i*-th germplasm, and *X*_*j*max_ and *X*_*j*min_ denote the maximum and minimum values of the *j*-th indicator, respectively. The result *Y_ij_* is the normalized value.

PCA was then performed on the normalized data to reduce dimensionality and extract major components. A comprehensive score for each germplasm resource was calculated using:

[2] *F*(*Y_ij_*) = *a*_1*j*_*Y*_1*j*_ + *a*_2*j*_*Y*_2*j*_ + . . . + *a_ij_Y_ij_*(*i* = 1,2,. . .,*n*; *j* = 1,2,. . .,*n*).

where *a_ij_* is the loading (eigenvector coefficient) of the *j*-th variable on the *i*-th principal component.

Subsequently, the weight *W_j_* of each principal component was calculated using:

[3] *W_j_* = *P_j_*/∑*P_j_*(*j* = 1,2,3,. . .,*n*).

where *P_j_* represents the contribution rate (explained variance) of the *j*-th principal component.

Finally, the comprehensive drought resistance score (*D*-value) for each tea plant germplasm resource was calculated as:

[4] *D* = ∑*F*(*Y_ij_*) × *W_j_*(*j* = 1,2,3,. . .,*n*).

A higher *D*-value indicates a stronger drought resistance capacity.

### Whole-genome resequencing

Genomic DNA was isolated from the third to fifth mature leaves below the apical bud of 107 tea plant cultivar/variety via CTAB extraction [61]. For the remaining eight tea accessions, publicly available raw sequencing data were downloaded from the NCBI Sequence Read Archive (PRJNA597714 and PRJNA665594) as reported by Kong *et al.* [21]. After quality assessment, the qualified DNA was used for library preparation. Whole genome sequencing (WGS) was performed on the Illumina NovaSeq 6000 platform, achieving an average sequencing depth of 10×. Raw sequencing reads were subjected to quality control. Reads were discarded if they contained adapter sequences, had a high proportion (>50%) of low-quality bases (*Q*-score ≤ 19), or contained more than 5% undetermined bases (N). The resulting high-quality Clean Reads were aligned to the tea reference genome ‘Lucha 6’, a chromosome-level genome assembled by our research group, using BWA software. Reads that were unmapped, of low mapping quality (MQ < 4), or marked as duplicates were removed. Subsequently, GATK was used to identify putative SNPs across all samples. SNPs were filtered according to the following criteria: QD < 2.0, ReadPosRankSum < −8.0, FS > 60.0, QUAL <30.0, DP < 4.0, SOR > 3.0, MQ < 40.0, and MQRankSum < −12.5. Finally, PLINK v1.9 was used to perform quality control on the retained SNP dataset, and GEC software [62] was applied to estimate the effective number of SNPs for subsequent GWAS.

### Population structure and LD

PCA of the tea plant population was performed based on SNP data using EIGENSOFT software. Population structure was inferred using ADMIXTURE software, with the number of ancestral populations (K) set from 2 to 10. The optimal K value was determined based on the cross-validation (CV) error, where the K with the lowest CV error was selected. A population structure plot was subsequently generated. A phylogenetic tree was constructed using the neighbour-joining method implemented in PHYLIP software, and visualized using the Interactive Tree Of Life (iTOL) online tool (https://itol.embl.de/). LD analysis was conducted using the PopLDdecay tool. The average LD coefficient (*r*^2^) was calculated, and an LD decay plot was generated. In the plot, the *x*-axis represents physical distance (bp), and the *y*-axis represents the corresponding average LD coefficient (*r*^2^) at that distance.

### GWAS

Genome-wide association analysis was conducted using the GEMMA software, based on a LMM. The analysis was performed to identify associations between SNP markers and 11 drought-related traits, as well as the comprehensive evaluation index (*D*-value), across the tea plant population. The population structure (Q matrix), derived from PCA, was included as a fixed effect. The kinship matrix (K matrix) was incorporated as a random effect to account for genetic relatedness among individuals [63]. The results of the GWAS were visualized using Manhattan plots and Q-Q plots. SNP loci were considered significantly associated with drought resistance if the *P*-value < α = 0.05 / number of valid SNPs, and suggestively associated if the *P*-value < α = 0.1 / number of valid SNPs.

### Candidate gene screening and bioinformatic analysis

Candidate regions were defined as 50 kb upstream and downstream of SNP loci significantly associated with each trait. Within these regions, key genes potentially associated with drought resistance were identified. Conduct a systematic bioinformatics functional analysis of the candidate genes [64]. These analyses included assessments of protein properties, chromosomal localization, gene structure, and cis-acting elements in the promoter regions.

### Transient silencing and overexpression of CsAGD6 in tea plant

Transient gene silencing was performed using antisense oligonucleotide (AsODN) technology. Sterile water was used as the sODN control. Ten μM AsODN primer solution and sterile water were injected into the mature leaves of one-year-old clonal tea plant cultivar ‘Baiye 1’ and ‘Longjing 43’. 10% PEG-6000 solution was applied to simulate drought stress. Leaf samples were collected at 0, 1, 3, and 5 days after treatment to assess drought resistance-related physiological indicators. The relevant primers are listed in Table S12. Each treatment included three biological replicates, and each replicate consisted of 10 seedlings.

Based on the method described [65], the *CsAGD6* gene was cloned into the overexpression vector pBTEX using Gateway technology, and the recombinant plasmid was introduced into *Agrobacterium tumefaciens* strain GV3101. The GV3101 strains harbouring either the empty vector or the *CsAGD6* overexpression construct were infiltrated into the mature leaves of one-year-old clonal tea plant cultivar ‘Baiye 1’ and ‘Longjing 43’. After 24 h of dark treatment, the plants were transferred to normal light conditions, and 10% PEG-6000 was applied to simulate drought stress. Samples were collected at 0, 1, 3, and 6 days post-treatment to evaluate drought resistance-related physiological indicators. Each treatment included three biological replicates, with 10 seedlings per replicate. The primer sequences used for vector construction are listed in Table S13.

### Subcellular localization of *CsAGD6* in *Nicotiana benthamiana*

Subcellular localization vectors were also constructed using Gateway technology. Agrobacterium strains containing either the pCAMBia1300-35S-EGFP vector or pCAMBia1300-35S-CsAGD6-EGFP were infiltrated into tobacco leaves. After 24 h of dark incubation, GFP fluorescence was observed using a laser scanning confocal microscope. The primer sequences used for vector construction are listed in Table S13.

### Haplotype analysis

Haplotype identification and network analysis were performed using the geneHapR [66] package in R, based on resequencing data from 287 tea plant accessions maintained by our research group. The geographical distribution of the major haplotypes was visually analyzed after being downloaded from the official standard map website of China (http://bzdt.ch.mnr.gov.cn/index.html) and the associations between haplotypes and phenotypic traits were analyzed using GraphPad Prism 8. Haplotypes represented by fewer than five individuals were considered rare and excluded from further analysis.

### RNA-seq and qRT-PCR analysis

Different tissues (flower buds, mature stems, mature leaves, green stems, and roots) from one-year-old ‘Shuchazao’ cutting seedlings, together with germplasm resources from different haplotypes, were used as materials for qRT-PCR [67] verification. The third to fifth mature leaves were collected from the drought-resistant cultivar ‘Longjing 43’ and the drought-sensitive cultivar ‘Baiye 1’ before and after drought stress for transcriptome sequencing and quantitative analysis. The total RNA of all above mentioned samples were extracted using the RNA extraction kit (All-purpose plant RNA Extraction Kit, China Kangwei Century) for RNA-seq and qRT-PCR analysis. Conduct RNA-seq and differential gene screening [67]. In addition, the same steps were also carried out to extract RNA for the varieties under different haplotypes screened out. The tea plant polypyrimidine channel binding protein gene (*CsPTB*) was used as the internal reference gene [68]. Relative gene expression levels were calculated using the 2^−ΔCt^ or 2^−ΔΔCt^ method [69]. Each cDNA template was analyzed in triplicate technical replicates, and results were expressed as mean ± standard error (±SE). Primer sequences used for qRT-PCR are listed in Table S14.

### Genotyping

Genomic DNA was extracted from 104 tea accessions, including 53 with known drought resistance from the 115 tea accessions GWAS panel and 51 set is independent with unknown resistance status and not part of the 115 tea accessions, using the FastPure Plant DNA Isolation Mini Kit (Novizan, Nanjing, China). Genotyping was performed as follows: the flanking sequences of candidate SNP loci were retrieved from the reference genome of cultivar ‘Lucha 6’, and corresponding primers were designed based on the principles of KASP.

KASP reactions (240 μl total volume) contained the following components: 1 μl of 2× KASP Master Mix (Chengdu Hanchen Guangyi Technology Co., Ltd., Chengdu, China), 0.004 μl each of two allele-specific forward primers, and 0.012 μl of a common reverse primer. After thorough mixing, the mixture was transferred into 200-μl centrifuge tubes. A 96-sample × 4-locus PCR system was assembled using a Matrix Arrayer fully automated liquid workstation, with a total reaction volume of 2 μl, including 1 μl of DNA template per reaction.

### Statistical analysis

Statistical analyses were conducted using SPSS 18.0 software. One-way ANOVA was employed to assess differences between groups, and Duncan’s multiple range test was used for *post hoc* comparisons. Descriptive statistics (including minimum, maximum, mean, median, standard deviation, and coefficient of variation), PCA, correlation analysis, and normality testing were also performed in SPSS18.0 software. Graphs, including box plots, linear regression plots, and phenotypic association charts, were generated using GraphPad Prism 8.

## Supplementary Material

Web_Material_uhaf320

## Data Availability

The transcriptome data and original sequencing of 107 tea accessions were deposited in the NCBI Sequence Read Archive database under accession numbers PRJNA1297129, PRJNA1304154 and PRJNA1314853. The tea reference genome ‘Lucha 6’, was deposited in the National Genomics Data Centre database (https://ngdc.cncb.ac.cn/gwh) under the BioProject is PRJCA049695 and accession number GWHGZIG00000000.1. The data that support the results are provided in this paper and its supplementary files.
