# Supplementary material for: Genome-wide association study and functional validation of CsAGD6 conferring drought tolerance in tea plant
Source: Hortic Res. 2025 Nov 21;13(3):uhaf320. doi: 10.1093/hr/uhaf320 (PMC12981331; doi:10.1093/hr/uhaf320)
Supplement: Web_Material_uhaf320 [file web_material_uhaf320.zip › Supplementary Figure.docx]

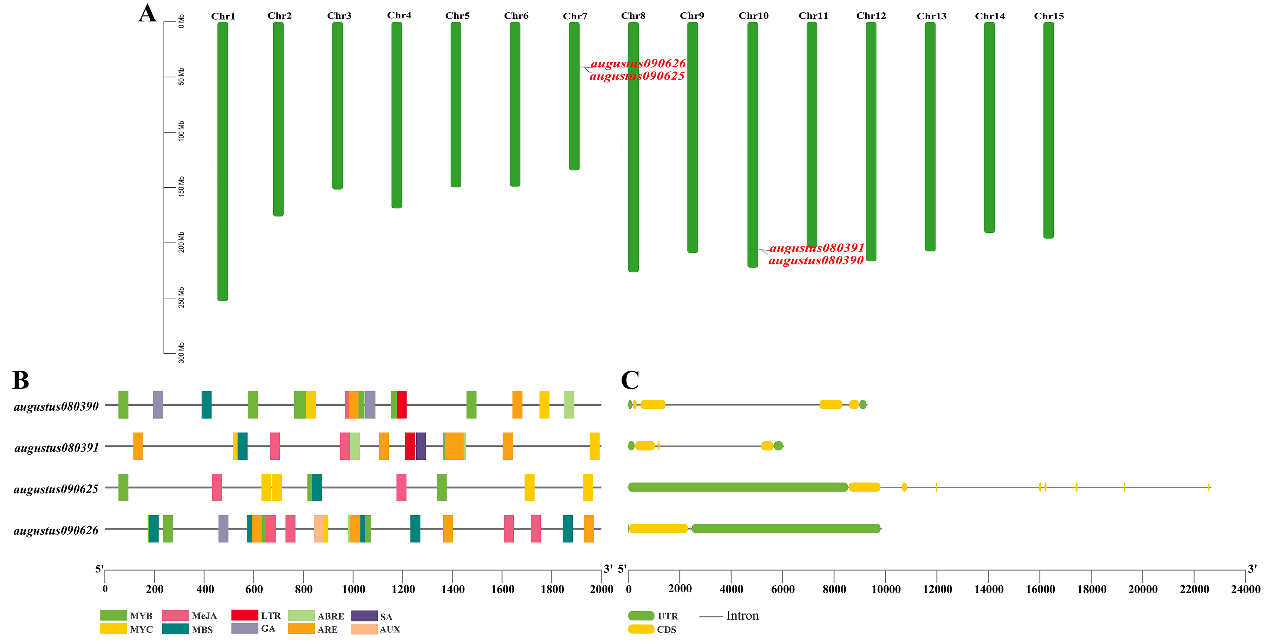


**Fig. S1. Chromosomal localization, promoter cis-element prediction, and gene structure analysis of candidate genes.** (A) Chromosomal localization; B) Promoter cis-element analysis; C) Gene structure visualization.


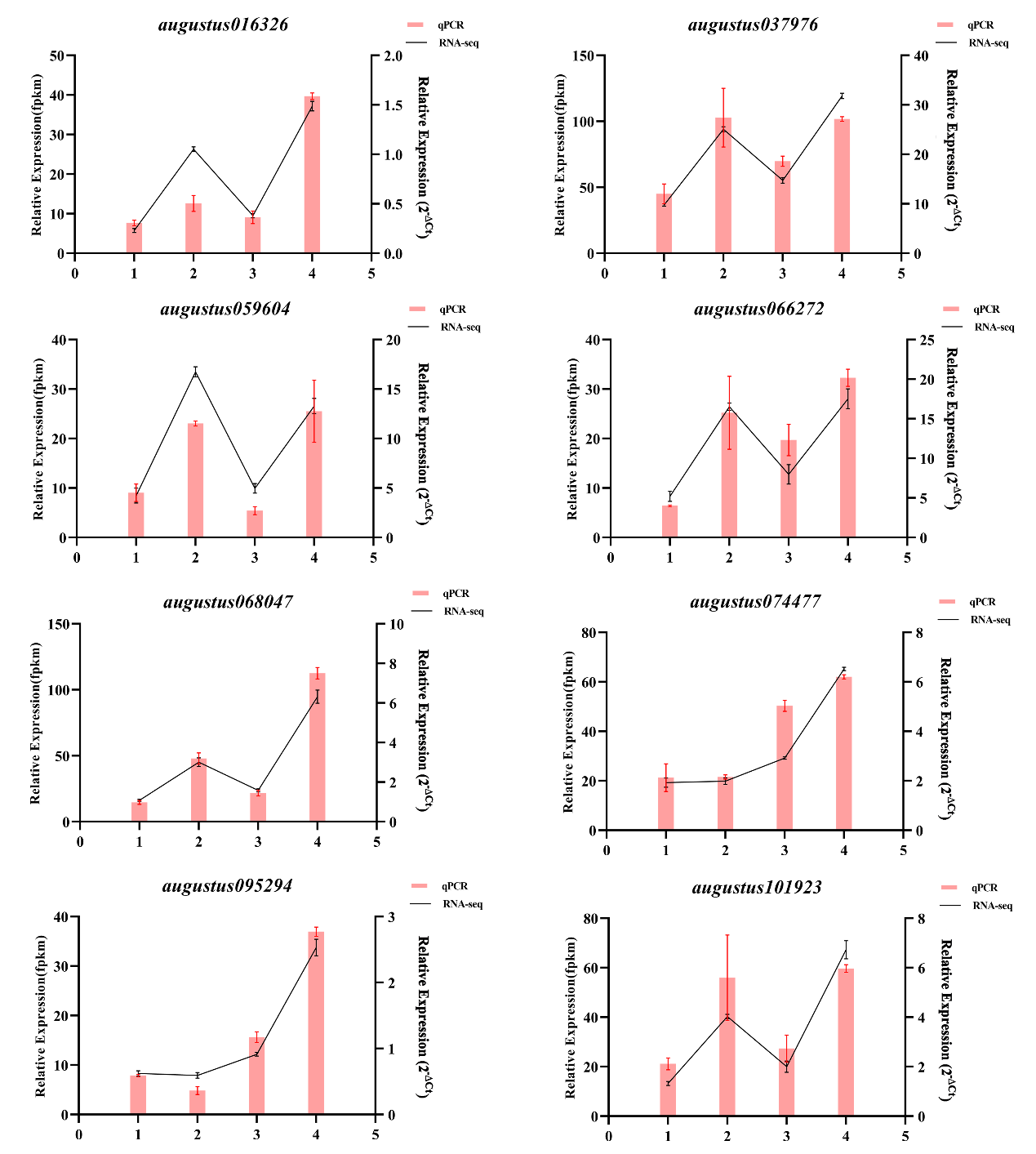


**Fig. S2. Comparison of qRT-PCR and RNA-seq expression data of candidate genes.**


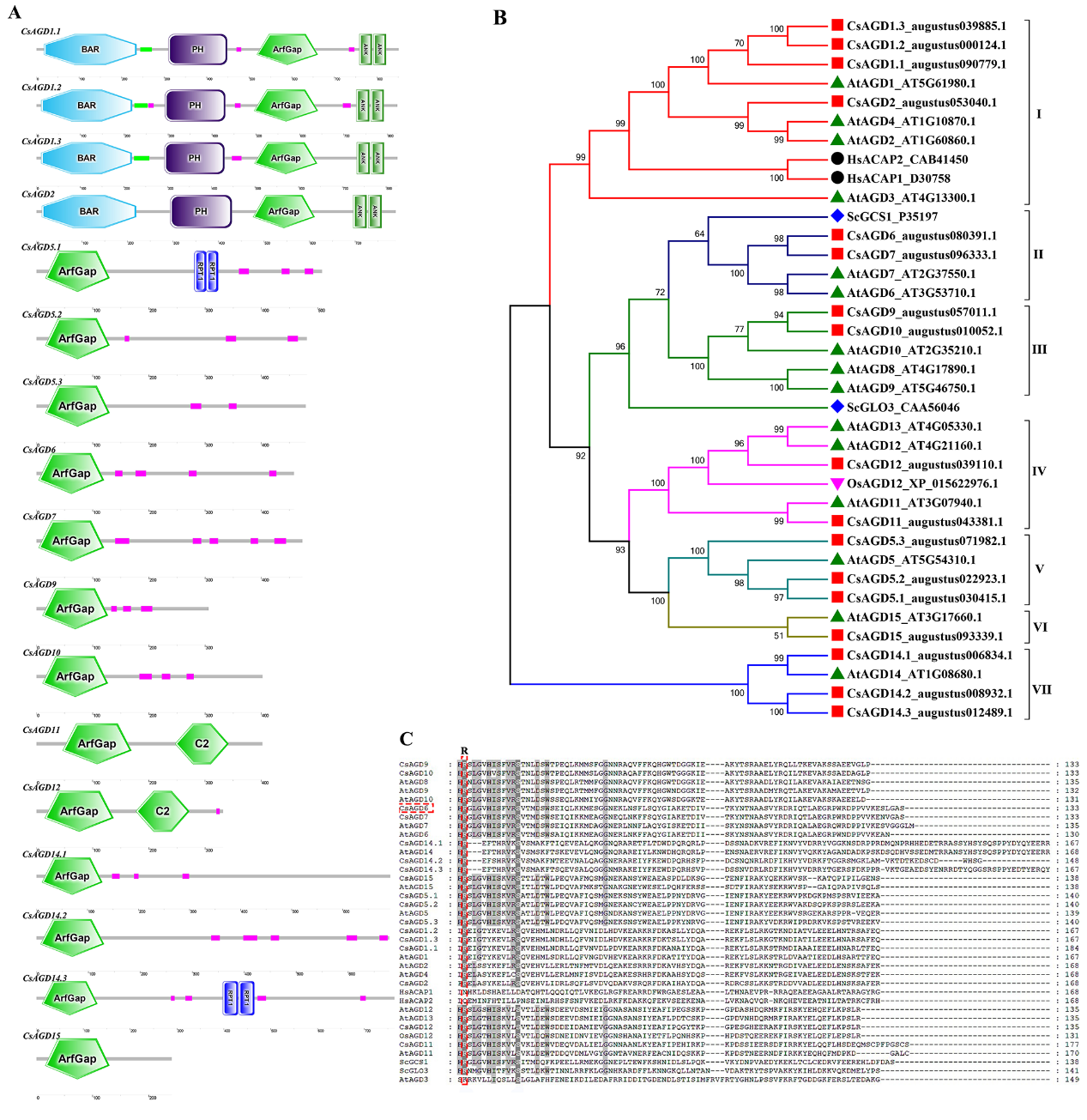


**Fig. S3. Analysis of the CsAGD gene family.** (A) Conserved GAP domains; B) Phylogenetic tree of CsAGD proteins; C) Multiple sequence alignment of amino acid sequences.


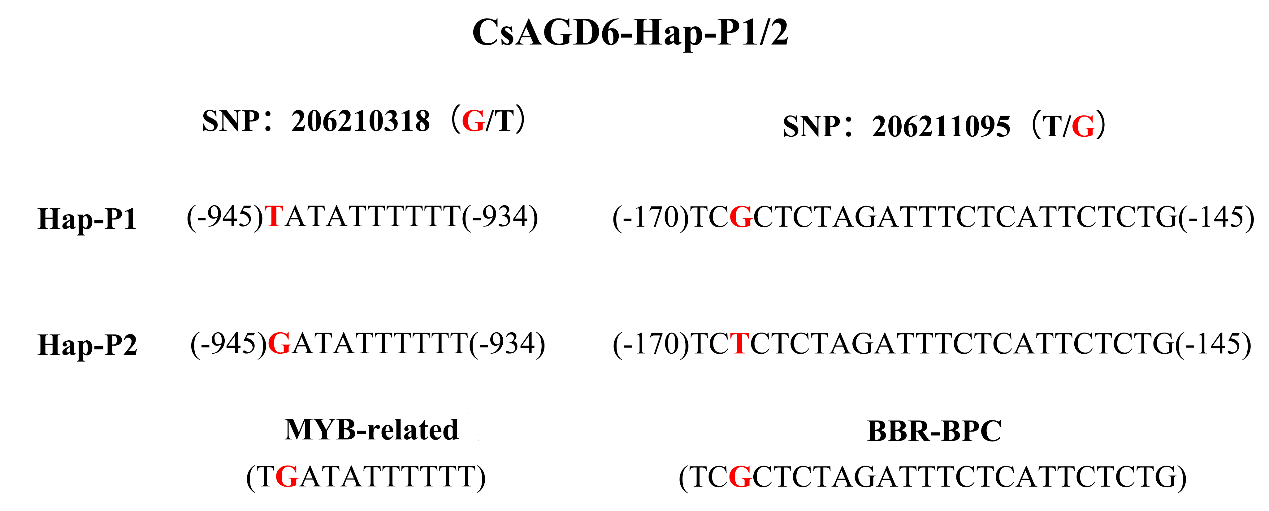


**Fig. S4. The mutation site in the *CsAGD6* promoter region**.


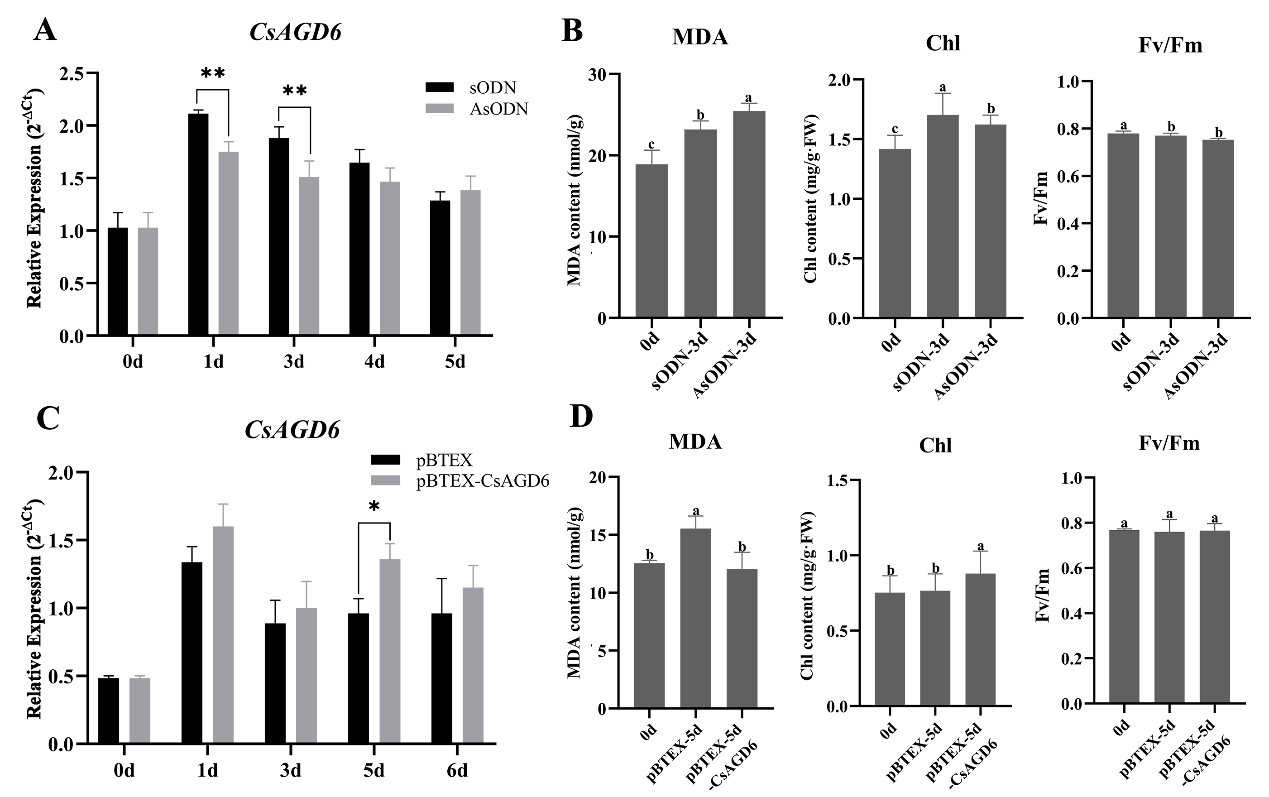


**Fig.S5. Transient functional validation of *CsAGD6* under drought stress in LongJing43.**

(A) Relative expression level of *CsAGD6* after transient gene silencing using antisense oligonucleotides (AsODN); (B) Physiological measurements in CsAGD6-silenced tea leaves after drought stress. (C) Relative expression level of CsAGD6 in tea leaves transiently overexpressing *CsAGD6*. (D) Drought-related physiological indices in CsAGD6-overexpressing plants. Different letters indicate significant differences at P < 0.05 based on ANOVA followed by Tukey’s test.
