# Supplementary material for: Genome-wide association study and functional validation of CsAGD6 conferring drought tolerance in tea plant
Source: Hortic Res. 2025 Nov 21;13(3):uhaf320. doi: 10.1093/hr/uhaf320 (PMC12981331; doi:10.1093/hr/uhaf320)
Supplement: Web_Material_uhaf320 [file web_material_uhaf320.zip › Supplementary Table.docx]

**Supplementary**

**Table S1. Variation and principal component analysis of drought resistance indices in tea plants**

| **Physiological indicators** | **Minimum value** | **Maximum value** | **Average value** | **Coefficient of Variation** | **Factor loading** | | |
| --- | --- | --- | --- | --- | --- | --- | --- |
|  |  |  |  |  | **PC1** | **PC2** | **PC3** |
| Pn | 0.7081 | 6.7805 | 3.5704 | 31.7 | 0.769 | 0.211 | 0.296 |
| Cond | 0.0063 | 0.0473 | 0.0194 | 41.3 | 0.497 | 0.581 | -0.255 |
| Trmmol | 0.1081 | 0.8346 | 0.3448 | 39.6 | 0.539 | 0.730 | -0.213 |
| WUE | -3.0412 | 8.3681 | 1.5898 | 71.3 | 0.339 | -0.490 | 0.701 |
| Fv/Fm | 0.0006 | 0.2883 | 0.0655 | 79.9 | 0.709 | 0.231 | 0352 |
| RWC | 0.2394 | 15.9298 | 5.2439 | 74.7 | 0.793 | 0.219 | 0.180 |
| MDA  Chl a  Chl b  Chl  Car | 1.2569 | 22.2530 | 7.3222 | 54.0 | 0.563 | 0.147 | 0.576 |
|  | 0.0175 | 0.6125 | 0.1814 | 67.9 | 0.847 | -0.273 | -0.332 |
|  | 0.0012 | 0.2444 | 0.0790 | 70.8 | 0.653 | -0.455 | -0.177 |
|  | 0.0186 | 0.8492 | 0.2532 | 66.3 | 0.867 | -0.369 | -0.299 |
|  | 0.0004 | 0.2112 | 0.0566 | 75.0 | 0.860 | -0.292 | -0.331 |
| **Eigenvalue** |  | | | | 5.327 | 1.781 | 1.517 |
| **Variance contribution rate** |  |  |  |  | 48.432 | 16.194 | 13.792 |
| **Cumulative contribution rate** |  |  |  |  | 48.432 | 64.625 | 78.417 |
| **Factor weight** |  |  |  |  | 0.618 | 0.207 | 0.176 |

Note: Pn: Net photosynthetic rate; Cond: Porosity; Ci: Intercellular CO2 concentration; Trmmol: Transpiration rate; WUE: Water Use Efficiency; Fv/Fm: Chlorophyll fluorescence parameter; MDA: Malondialdehyde; Chl a: Chlorophyll a; Chl b: Chlorophyll b; Chl: Chlorophyll; Car: Carotenoids; PC1-3 represent the first to the third principal components respectively.

**Table S2. Drought resistance ranking of 115 tea germplasm resources**

| ID | Germplasm name | D | Sorting | ID | Germplasm name | D | Sorting |
| --- | --- | --- | --- | --- | --- | --- | --- |
| A253 | MengShan 9 | 4.444 | 1 | A74 | LuCha12 | 3.316 | 59 |
| A266 | ZheNong 117 | 4.313 | 2 | A65 | JiuKeng 1 | 3.310 | 60 |
| A262 | JinGuanYin | 4.232 | 3 | A7 | ECha 1 | 3.304 | 61 |
| A255 | XinYang 10 | 4.215 | 4 | A228 | YuJinXiang | 3.303 | 62 |
| A47 | D11 | 4.149 | 5 | A96 | LuCha31 | 3.297 | 63 |
| A288 | MaBianLv | 4.085 | 6 | A112 | ZiJuan | 3.286 | 64 |
| A44 | D1 | 4.079 | 7 | A220 | BanTianYao | 3.274 | 65 |
| A62 | PingYangTeZao | 4.018 | 8 | A85 | LuCha34 | 3.246 | 66 |
| A43 | D15 | 4.013 | 9 | A233 | TeZao 213 | 3.228 | 67 |
| A48 | D5 | 3.997 | 10 | A240 | LuCha38 | 3.216 | 68 |
| A40 | D12 | 3.976 | 11 | A279 | ChuanJiu | 3.141 | 69 |
| A218 | DaHongPao | 3.964 | 12 | A6 | ShanCha 1 | 3.132 | 70 |
| A263 | ShuChaZao | 3.951 | 13 | A194 | LuCha28 | 3.125 | 71 |
| A23 | D4 | 3.939 | 14 | A184 | LongJingChangYe | 3.102 | 72 |
| A189 | LuCha 19 | 3.926 | 15 | A154 | JinGuan | 3.090 | 73 |
| A280 | XiaoYeJiBai | 3.883 | 16 | A179 | NaiBai | 3.084 | 74 |
| A80 | LuCha20 | 3.873 | 17 | A236 | LuCha39 | 3.083 | 75 |
| A69 | ZiMuDan | 3.872 | 18 | A133 | MingShan 131 | 3.064 | 76 |
| A86 | LuCha21 | 3.868 | 19 | A275 | ZhongHuang 1 | 3.055 | 77 |
| A285 | ZheNong 113 | 3.843 | 20 | A237 | LuCha 40 | 3.033 | 78 |
| A277 | TeZaoBai | 3.831 | 21 | A83 | LuCha 1 | 3.032 | 79 |
| A286 | JianYeNaiBai | 3.827 | 22 | A244 | LuCha 5 | 3.032 | 80 |
| A197 | LuMing4 | 3.795 | 23 | A19 | HuangJinYe | 3.023 | 81 |
| A88 | LuCha 22 | 3.792 | 24 | A190 | LianShan 1 | 3.011 | 82 |
| A138 | ZhongHuang 2 | 3.784 | 25 | A22 | ShuYong 307 | 2.970 | 83 |
| A73 | LuCha24 | 3.781 | 26 | A16 | FuDingDaHao | 2.927 | 84 |
| A274 | FuDingDaBai | 3.779 | 27 | A11 | ChuanMu 28 | 2.906 | 85 |
| A41 | D14 | 3.760 | 28 | A281 | BaiZhenZhu | 2.878 | 86 |
| A99 | LuCha37 | 3.759 | 29 | A199 | LuCha13 | 2.838 | 87 |
| A245 | LuCha29 | 3.750 | 30 | A87 | LuCha 7 | 2.813 | 88 |
| A72 | JiuKeng 2 | 3.733 | 31 | A75 | LuCha17 | 2.761 | 89 |
| A180 | LuMing9 | 3.726 | 32 | A113 | FuXuan 9 | 2.742 | 90 |
| A252 | SuChaZao | 3.711 | 33 | A123 | HuangJinYa | 2.734 | 91 |
| A111 | ZiMeiGui | 3.706 | 34 | A238 | LuCha 14 | 2.702 | 92 |
| A287 | ZaoHuangHuang | 3.703 | 35 | A198 | LuCha 4 | 2.628 | 93 |
| A282 | BaiYuXian | 3.702 | 36 | A134 | FuYun 6 | 2.602 | 94 |
| A186 | ZhongHuang 3 | 3.695 | 37 | A46 | D2 | 2.587 | 95 |
| A260 | HuangGuanYin | 3.647 | 38 | A178 | RouGui | 2.574 | 96 |
| A203 | LuCha 15 | 3.637 | 39 | A201 | LuCha 26 | 2.560 | 97 |
| A261 | HuangMeiGui | 3.617 | 40 | A230 | BaiYe 1 | 2.556 | 98 |
| A132 | SuCha 120 | 3.600 | 41 | A243 | LuCha 36 | 2.537 | 99 |
| A246 | LuCha 27 | 3.557 | 42 | A20 | ShuYong 703 | 2.445 | 100 |
| A241 | LuCha 16 | 3.522 | 43 | A267 | ZhongCha 102 | 2.427 | 101 |
| A149 | LuCha 18 | 3.518 | 44 | A160 | DaGangZhong | 2.399 | 102 |
| A221 | ShuiXian | 3.513 | 45 | A278 | WenChun | 2.396 | 103 |
| A276 | TianLaoHuang | 3.507 | 46 | A13 | ChuanCha 2 | 2.262 | 104 |
| A239 | LuCha 30 | 3.504 | 47 | A283 | wufeng 212 | 2.245 | 105 |
| A290 | ShuiJingBai | 3.490 | 48 | A121 | MengShan 4 | 2.204 | 106 |
| A78 | LuCha 33 | 3.434 | 49 | A251 | TaiCha 12 | 2.203 | 107 |
| A77 | LuCha 32 | 3.425 | 50 | A264 | TieLuoHan | 2.125 | 108 |
| A289 | BaiJinYa | 3.424 | 51 | A45 | D13 | 2.044 | 109 |
| A185 | ZhongBai 1 | 3.418 | 52 | A268 | ZhongCha 108 | 1.943 | 110 |
| A68 | QingXinWuLong | 3.389 | 53 | A4 | WuNiuZao | 1.883 | 111 |
| A270 | LongJing 43 | 3.378 | 54 | A120 | ZhongCha 111 | 1.808 | 112 |
| A192 | LuCha35 | 3.375 | 55 | A10 | JinMuDan | 1.657 | 113 |
| A214 | LuMing3 | 3.365 | 56 | A5 | MingShanZao 311 | 1.043 | 114 |
| A226 | NongKangZao | 3.338 | 57 | A284 | YiChangDaYeZhong | 1.022 | 115 |
| A211 | LuCha 11 | 3.318 | 58 |  |  |  |  |

**Table S3. Summary of associated SNP loci**

| Trait | SNP | CHR | Location(bp) | Location | *P* |
| --- | --- | --- | --- | --- | --- |
| RWC | chr5:65128905 | 5 | 65128905 | Intergene region | 1.67E-09 |
| MDA | chr7:64407629 | 7 | 64407629 | Intergene region | 3.87E-10 |
| MDA | chr13:130485628 | 13 | 130485628 | Intergene region | 1.45E-09 |
| MDA | chr14:162152124 | 14 | 162152124 | Intergene region | 1.62E-09 |
| MDA | chr1:119189840 | 1 | 119189840 | Intergene region | 2.31E-09 |
| MDA | chr1:119189841 | 1 | 119189841 | Intergene region | 2.31E-09 |
| MDA | chr11:37231843 | 11 | 37231843 | Intergene region | 3.52E-09 |
| Pn | chr6:148468093 | 6 | 148468093 | Intergene region | 2.63E-09 |
| Cond | chr6:109207426 | 6 | 109207426 | Intergene region | 1.00E-09 |
| Cond | chr14:159011958 | 14 | 159011958 | Intergene region | 1.49E-09 |
| Cond | chr3:67772003 | 3 | 67772003 | Intergene region | 3.24E-09 |
| Trmmol | chr10:42288509 | 10 | 42288509 | Intergene region | 2.32E-09 |
| Chl a | chr3:64761481 | 3 | 64761481 | Intergene region | 2.85E-10 |
| Chl a | chr15:157502868 | 15 | 157502868 | Intergene region | 1.26E-09 |
| Chl a | chr9:76677440 | 9 | 76677440 | Intergene region | 1.84E-09 |
| Chl a | chr12:25026763 | 12 | 25026763 | Intergene region | 2.81E-09 |
| Chl b | chr10:121211775 | 10 | 121211775 | Intergene region | 3.98E-10 |
| Chl b | chr11:130671257 | 11 | 130671257 | Intergene region | 8.70E-10 |
| Chl b | chr15:163694942 | 15 | 163694942 | Intergene region | 1.33E-09 |
| Chl b | chr1:185613340 | 1 | 185613340 | Intergene region | 2.54E-09 |
| Chl | chr9:76677440 | 9 | 76677440 | Intergene region | 1.45E-09 |
| Car | chr15:162270855 | 15 | 162270855 | Intergene region | 6.09E-10 |
| Car | chr15:157502868 | 15 | 157502868 | Intergene region | 1.61E-09 |
| Car | chr1:192653275 | 1 | 192653275 | Intergene region | 2.01E-09 |
| Car | chr11:130671257 | 11 | 130671257 | Intergene region | 2.48E-09 |
| Car | chr9:76677440 | 9 | 76677440 | Intergene region | 3.27E-09 |
| Fv/Fm | chr10:206112846 | 10 | 206112846 | Intergene region | 5.26E-14 |
| Fv/Fm | chr10:206213091 | 10 | 206213091 | Intron region | 4.68E-12 |
| Fv/Fm | chr10:206157860 | 10 | 206157860 | Intergene region | 1.68E-11 |
| Fv/Fm | chr3:62472747 | 3 | 62472747 | Intergene region | 5.86E-11 |
| Fv/Fm | chr10:206214174 | 10 | 206214174 | Intron region | 6.52E-11 |
| Fv/Fm | chr10:206216595 | 10 | 206216595 | Exon region | 6.55E-11 |
| Fv/Fm | chr10:206216490 | 10 | 206216490 | Exon region | 7.02E-11 |
| Fv/Fm | chr10:206216511 | 10 | 206216511 | Exon region | 7.02E-11 |
| Fv/Fm | chr10:206206063 | 10 | 206206063 | Intergene region | 7.04E-11 |
| Fv/Fm | chr10:206216592 | 10 | 206216592 | Exon region | 8.72E-11 |
| Fv/Fm | chr10:206216643 | 10 | 206216643 | Exon region | 8.72E-11 |
| Fv/Fm | chr10:206216660 | 10 | 206216660 | Exon region | 8.72E-11 |
| Fv/Fm | chr10:206213524 | 10 | 206213524 | Intron region | 9.44E-11 |
| Fv/Fm | chr10:206214151 | 10 | 206214151 | Intron region | 1.20E-10 |
| Fv/Fm | chr10:206139240 | 10 | 206139240 | Intergene region | 1.30E-10 |
| Fv/Fm | chr10:206216541 | 10 | 206216541 | Exon region | 1.41E-10 |
| Fv/Fm | chr10:206156684 | 10 | 206156684 | Intergene region | 1.57E-10 |
| Fv/Fm | chr10:206174109 | 10 | 206174109 | Intergene region | 2.69E-10 |
| Fv/Fm | chr3:62472708 | 3 | 62472708 | Intergene region | 2.91E-10 |
| Fv/Fm | chr2:49897112 | 2 | 49897112 | Intergene region | 2.92E-10 |
| Fv/Fm | chr10:206214617 | 10 | 206214617 | Intron region | 3.36E-10 |
| Fv/Fm | chr10:206215559 | 10 | 206215559 | Intron region | 3.36E-10 |
| Fv/Fm | chr10:206213638 | 10 | 206213638 | Intron region | 3.79E-10 |
| Fv/Fm | chr10:206216283 | 10 | 206216283 | Exon region | 4.41E-10 |
| Fv/Fm | chr10:206156687 | 10 | 206156687 | Intergene region | 4.62E-10 |
| Fv/Fm | chr10:206151292 | 10 | 206151292 | Intergene region | 6.48E-10 |
| Fv/Fm | chr10:206214348 | 10 | 206214348 | Intron region | 7.41E-10 |
| Fv/Fm | chr10:206214920 | 10 | 206214920 | Intron region | 1.02E-09 |
| Fv/Fm | chr10:206215323 | 10 | 206215323 | Intron region | 1.18E-09 |
| Fv/Fm | chr10:206215945 | 10 | 206215945 | Intron region | 1.18E-09 |
| Fv/Fm | chr10:206215985 | 10 | 206215985 | Intron region | 1.18E-09 |
| Fv/Fm | chr10:206212213 | 10 | 206212213 | Exon region | 1.26E-09 |
| Fv/Fm | chr10:206213231 | 10 | 206213231 | Intron region | 1.26E-09 |
| Fv/Fm | chr10:206213786 | 10 | 206213786 | Intron region | 1.26E-09 |
| Fv/Fm | chr10:206213994 | 10 | 206213994 | Intron region | 1.26E-09 |
| Fv/Fm | chr10:206215856 | 10 | 206215856 | Intron region | 1.26E-09 |
| Fv/Fm | chr10:206216217 | 10 | 206216217 | Exon region | 1.26E-09 |
| Fv/Fm | chr10:206216451 | 10 | 206216451 | Exon region | 1.26E-09 |
| Fv/Fm | chr10:206218004 | 10 | 206218004 | UTR3 | 1.26E-09 |
| Fv/Fm | chr4:91698610 | 4 | 91698610 | Intergene region | 1.35E-09 |
| Fv/Fm | chr10:206214434 | 10 | 206214434 | Intron region | 1.43E-09 |
| Fv/Fm | chr10:206213247 | 10 | 206213247 | Intron region | 1.54E-09 |
| Fv/Fm | chr10:206215096 | 10 | 206215096 | Intron region | 1.58E-09 |
| Fv/Fm | chr10:206214313 | 10 | 206214313 | Intron region | 1.71E-09 |
| Fv/Fm | chr10:206214670 | 10 | 206214670 | Intron region | 1.75E-09 |
| Fv/Fm | chr10:206109317 | 10 | 206109317 | Intergene region | 1.84E-09 |
| Fv/Fm | chr10:206217150 | 10 | 206217150 | Within 1000 bp downstream of the gene | 2.19E-09 |
| Fv/Fm | chr10:206165939 | 10 | 206165939 | Intergene region | 2.35E-09 |
| Fv/Fm | chr10:206215148 | 10 | 206215148 | Intron region | 2.51E-09 |
| Fv/Fm | chr10:206215068 | 10 | 206215068 | Intron region | 2.58E-09 |
| Fv/Fm | chr11:106339469 | 11 | 106339469 | Intron region | 2.97E-09 |
| Fv/Fm | chr10:206215817 | 10 | 206215817 | Intron region | 2.99E-09 |
| Fv/Fm | chr7:26550509 | 7 | 26550509 | Intergene region | 3.51E-09 |

**Table S4. Candidate genes associated with drought resistance**

| Trait | Chr | Location interval | Candidate gene | Note |
| --- | --- | --- | --- | --- |
| Fv/Fm | 10 | 206218031-206226843 | *CsNHX1* | Cation /H (+) reverse transporter |
| Fv/Fm | 10 | 206211262-206216661 | *CsAGD6* | ADP ribosylation factor GTPase activating protein AGD6 |
| MDA | 7 | 40917002-40931056 | *CsRPP13-LK3* | Disease-resistant RPP13-like protein 1 |
| MDA | 7 | 40937153-40939593 | *CsRPP13-LK4* | Disease-resistant RPP13-like protein 1 |

**Table S5. Physicochemical property analysis of candidate gene-encoded proteins**

| Candidate gene | Open reading box /bp | Amino acid length /aa | Molecular weight /kDa | Isoelectric point | Subcellular localization | Signal peptide | Transmembrane structure |
| --- | --- | --- | --- | --- | --- | --- | --- |
| *CsNHX1* | 2481 | 826 | 89.75 | 8.5 | Vacuole | No | Yes |
| *CsAGD6* | 1377 | 458 | 49.31 | 8.13 | Cell nucleus | No | No |
| *CsRPP13-LK3* | 2040 | 679 | 75.69 | 5.04 | Cell membrane | No | No |
| *CsRPP13-LK4* | 2367 | 788 | 89.48 | 6.56 | Cytoplasm | No | No |

**Table S6. Haplotype analysis of the *CsAGD6***

| SNP | Location | SNP | Location |
| --- | --- | --- | --- |
| Chr10:206209994 | Promoter region | Chr10:206210085 | Promoter region |
| Chr10:206210163 | Promoter region | Chr10:206210219 | Promoter region |
| Chr10:206210220 | Promoter region | Chr10:206210266 | Promoter region |
| Chr10:206210304 | Promoter region | Chr10:206210318 | Promoter region |
| Chr10:206210380 | Promoter region | Chr10:206210575 | Promoter region |
| Chr10:206211095 | Promoter region | Chr10:206211101 | Promoter region |
| Chr10:206211153 | Promoter region | Chr10:206211186 | Promoter region |
| Chr10:206211216 | Promoter region | Chr10:206211257 | Promoter region |
| Chr10:206211284 | Coding area | Chr10:206211312 | Coding area |
| Chr10:206211326 | Coding area | Chr10:206211365 | Coding area |
| Chr10:206211367 | Coding area | Chr10:206211436 | Coding area |
| Chr10:206211459 | Coding area | Chr10:206211493 | Coding area |
| Chr10:206211518 | Coding area | Chr10:206211538 | Coding area |
| Chr10:206211710 | Coding area | Chr10:206211869 | Coding area |
| Chr10:206216474 | Coding area | Chr10:206216490 | Coding area |
| Chr10:206216647 | Coding area |  |  |

**Table S7. Combined haplotype patterns**

| Promoter | CDS | Freq |
| --- | --- | --- |
| Hap-P1 | Hap-C1 | 0 |
| Hap-P1 | Hap-C2 | 7 |
| Hap-P2 | Hap-C1 | 1 |
| Hap-P2 | Hap-C2 | 0 |

**Table S8. SNP-KASP marker primer sequences**

| SNP | | Primer | Primer |
| --- | --- | --- | --- |
| chr10:206216541 | F-FAM  F-HEX  R | | GAAGGTGACCAAGTTCATGCTGGGATGGCTGGGCAGGC  GAAGGTCGGAGTCAACGGATTGGGATGGCTGGGCAGGT  CGAATCCATCATCCTTACCATCGTCC |

**Table S9. Genotyping results of 52 tea germplasm accessions.**

| Genotype | Germplasm name | D | Genotype | Germplasm name | D | Genotype | Germplasm name | D |
| --- | --- | --- | --- | --- | --- | --- | --- | --- |
| CC | ECha 1 | 3.304 | CC | ZheNong 113 | 3.843 | CC | ZheNong 117 | 4.313 |
| CC | LongJing 43 | 3.378 | CC | JiuKeng 2 | 3.733 | CC | ShuChaZao | 3.951 |
| CC | QingXinWuLong | 3.389 | CC | XinYang 10 | 4.215 | CC | HuangMeiGui | 3.617 |
| CC | DaHongPao | 3.964 | CC | ZiMuDan | 3.872 | CC | JinGuanYin | 4.232 |
| CC | HuangGuanYin | 3.647 | CC | ZiMeiGui | 3.706 | CC | MaBianLv | 4.085 |
| CC | PingYangTeZao | 4.018 | CC | SuChaZao | 3.711 | CC | NongKangZao | 3.338 |
| CC | BaiYuXian | 3.702 | CC | FuDingDaBai | 3.779 | CC | SuCha 120 | 3.600 |
| CC | LuMing9 | 3.726 | CC | LuCha 20 | 3.873 | CC | LuCha 21 | 3.868 |
| CC | ZhongCha 302 | 2.427 | CC | MengShan 9 | 4.444 | CC | ZhongHuang 2 | 3.784 |
| CC | TeZaoBai | 3.831 | CC | ZaoHuangHuang | 3.703 | CC | ZhongBai 1 | 3.418 |
| CC | ZhongHuang 3 | 3.695 | CC | LuCha 22 | 3.792 | CC | LuCha 24 | 3.781 |
| CC | LuCha 19 | 3.926 | CC | LuCha 37 | 3.759 | CT | FuDingDaHao | 2.927 |
| CT | ShanCha 1 | 3.132 | CT | JianYeNaiBai | 3.827 | CT | YiChangDaYeZhong | 1.022 |
| CT | ShuiXian | 3.513 | CT | BaiYe 1 | 2.556 | CT | WuFeng 212 | 2.245 |
| CT | LuCha 13 | 2.838 | CT | MengShan 4 | 2.204 | CT | HuangJinYa | 2.734 |
| CT | ChuanMu 28 | 2.906 | TT | WuNiuZao | 1.883 | TT | TaiCha 12 | 2.203 |
| TT | LuCha 36 | 2.537 | TT | ZhongCha 111 | 1.808 | TT | BaiZhenZhu | 2.878 |
| TT | TieLuoHan | 3.507 |  |  |  |  |  |  |

**Table S10. Physicochemical properties of CsAGD family proteins**

| Candidate gene | Open reading box /bp | Amino acid length /aa | Molecular weight /kDa | Isoelectric point | Signal peptide | Transmembrane structure |
| --- | --- | --- | --- | --- | --- | --- |
| *CsAGD1.1* | 2556 | 851 | 95.78 | 7.62 | No | No |
| *CsAGD1.2* | 2469 | 822 | 91.86 | 6.58 | No | No |
| *CsAGD1.3* | 2481 | 826 | 92.49 | 7.37 | No | No |
| *CsAGD2* | 2370 | 789 | 89.23 | 6.47 | No | No |
| *CsAGD5.1* | 1527 | 508 | 55.05 | 6.64 | No | No |
| *CsAGD5.2* | 1446 | 481 | 51.81 | 5.64 | No | No |
| *CsAGD5.3* | 1440 | 479 | 52.69 | 9.25 | No | No |
| *CsAGD6* | 1377 | 458 | 49.31 | 8.13 | No | No |
| *CsAGD7* | 1422 | 473 | 50.51 | 8.45 | No | No |
| *CsAGD9* | 921 | 306 | 32.94 | 9.34 | No | No |
| *CsAGD10* | 1209 | 402 | 43.23 | 8.9 | No | No |
| *CsAGD11* | 1209 | 402 | 44.84 | 6.47 | No | No |
| *CsAGD12* | 900 | 332 | 36.56 | 5.43 | No | No |
| *CsAGD14.1* | 2082 | 693 | 75.99 | 5.39 | No | No |
| *CsAGD14.2* | 2040 | 679 | 73.31 | 6.69 | No | No |
| *CsAGD14.3* | 2295 | 764 | 82.36 | 7.71 | No | No |
| *CsAGD15* | 726 | 241 | 27.09 | 8.9 | No | No |

**Table S11. Accession numbers and sources of 115 tea germplasm resources.**

| ID | Germplasm name | Geographical origin | ID | Germplasm name | Geographical origin |
| --- | --- | --- | --- | --- | --- |
| A226 | NongKangZao | Anhui | A270 | LongJing 43 | Zhejiang |
| A263 | ShuChaZao | Anhui | A275 | ZhongHuang 1 | Zhejiang |
| A10 | JinMuDan | Fujian | A276 | TianLaoHuang | Zhejiang |
| A16 | FuDingDaHao | Fujian | A277 | TeZaoBai | Zhejiang |
| A69 | ZiMuDan | Fujian | A280 | XiaoYeJiBai | Zhejiang |
| A111 | ZiMeiGui | Fujian | A281 | BaiZhenZhu | Zhejiang |
| A113 | FuXuan 9 | Fujian | A282 | BaiYuXian | Zhejiang |
| A134 | FuYun 6 | Fujian | A285 | ZheNong 113 | Zhejiang |
| A154 | JinGuan | Fujian | A286 | JianYeNaiBai | Zhejiang |
| A178 | RouGui | Fujian | A287 | ZaoHuangHuang | Zhejiang |
| A218 | DaHongPao | Fujian | A289 | BaiJinYa | Zhejiang |
| A220 | BanTianYao | Fujian | A290 | ShuiJingBai | Zhejiang |
| A221 | ShuiXian | Fujian | A23 | D4 | Shandong |
| A260 | HuangGuanYin | Fujian | A40 | D12 | Shandong |
| A261 | HuangMeiGui | Fujian | A41 | D14 | Shandong |
| A262 | JinGuanYin | Fujian | A43 | D15 | Shandong |
| A264 | TieLuoHan | Fujian | A44 | D1 | Shandong |
| A274 | FuDingDaBai | Fujian | A45 | D13 | Shandong |
| A255 | XinYang 10 | Henan | A46 | D2 | Shandong |
| A7 | ECha 1 | Hubei | A47 | D11 | Shandong |
| A283 | wufeng 212 | Hubei | A48 | D5 | Shandong |
| A284 | YiChangDaYeZhong | Hubei | A73 | LuCha 24 | Shandong |
| A132 | SuCha 120 | Jiangsu | A74 | LuCha 12 | Shandong |
| A252 | SuChaZao | Jiangsu | A75 | LuCha 17 | Shandong |
| A6 | ShanCha 1 | Shanxi | A77 | LuCha 32 | Shandong |
| A5 | MingShanZao 311 | Sichuan | A78 | LuCha 33 | Shandong |
| A11 | ChuanMu 28 | Sichuan | A80 | LuCha 20 | Shandong |
| A13 | ChuanCha 2 | Sichuan | A83 | LuCha 1 | Shandong |
| A20 | ShuYong 703 | Sichuan | A85 | LuCha 34 | Shandong |
| A22 | ShuYong 307 | Sichuan | A86 | LuCha 21 | Shandong |
| A121 | MengShan 4 | Sichuan | A87 | LuCha 7 | Shandong |
| A133 | MingShan 131 | Sichuan | A88 | LuCha 22 | Shandong |
| A233 | TeZao 213 | Sichuan | A96 | LuCha 31 | Shandong |
| A253 | MengShan 9 | Sichuan | A99 | LuCha 37 | Shandong |
| A278 | WenChun | Sichuan | A149 | LuCha 18 | Shandong |
| A279 | ChuanJiu | Sichuan | A180 | LuMing9 | Shandong |
| A288 | MaBianLv | Sichuan | A189 | LuCha 19 | Shandong |
| A68 | QingXinWuLong | Taiwan | A190 | LianShan 1 | Shandong |
| A251 | JinXuan | Taiwan | A192 | LuCha 35 | Shandong |
| A112 | ZiJuan | Yunnan | A194 | LuCha 28 | Shandong |
| A4 | WuNiuZao | Zhejiang | A197 | LuMing4 | Shandong |
| A19 | HuangJinYe | Zhejiang | A198 | LuCha 4 | Shandong |
| A62 | PingYangTeZao | Zhejiang | A199 | LuCha 13 | Shandong |
| A65 | JiuKeng 1 | Zhejiang | A201 | LuCha 26 | Shandong |
| A72 | JiuKeng 2 | Zhejiang | A203 | LuCha 15 | Shandong |
| A120 | ZhongCha 111 | Zhejiang | A211 | LuCha 11 | Shandong |
| A123 | HuangJinYa | Zhejiang | A214 | LnMing3 | Shandong |
| A138 | ZhongHuang 2 | Zhejiang | A236 | LuCha 39 | Shandong |
| A160 | DaGangZhong | Zhejiang | A237 | LuCha 40 | Shandong |
| A179 | NaiBai | Zhejiang | A238 | LuCha 14 | Shandong |
| A184 | LongJingChangYe | Zhejiang | A239 | LuCha 30 | Shandong |
| A185 | ZhongBai 1 | Zhejiang | A240 | LuCha 38 | Shandong |
| A186 | ZhongHuang 3 | Zhejiang | A241 | LuCha 16 | Shandong |
| A228 | YuJinXiang | Zhejiang | A243 | LuCha 36 | Shandong |
| A230 | BaiYe 1 | Zhejiang | A244 | LuCha 5 | Shandong |
| A266 | ZheNong 117 | Zhejiang | A245 | LuCha 29 | Shandong |
| A267 | ZhongCha 102 | Zhejiang | A246 | LuCha 27 | Shandong |
| A268 | ZhongCha 108 | Zhejiang |  |  |  |

**Table S12. AsODN primer sequences**

| Vector name | Primers |
| --- | --- |
| AsODN | TTCTTTTTGCTGGCGCCGAG |
|  | TGCAGTGACAACGTTGACCG |

**Table S13. Primer sequences for vector construction**

| Vector name | Forward/Reverse | Primers |
| --- | --- | --- |
| PBTEX  (HA) | F | ATTTGGAGAGGACAGGGTACCATGGCGGCATCGAGACGCCT |
|  | R | AACGTCGTATGGGTAAGGCCTGAAAAAGCCACTGTCACT |
| pCAMBia  1300-eGFP | F | CGAACGATAGCCATGGTACCAATGGCGGCATCGAGACGCCT |
|  | R | CATGCCTGCGGCCGCGCCGGATCCGAAAAAGCCACTGTCACT |

**Table S14. qRT-PCR primer sequences**

| Gene name | Sense primer (5´-→3´-) | Anti-sense primer (5´-→3´-) |
| --- | --- | --- |
| *CsPTB* | TGACCAAGCACACTCCACACTATCG | TGCCCCCTTATCATCATCCACAA |
| *augustus080390* | CCAGTCCTCGCTCGCATACTC | GACCGACACCAATGGGCTCT |
| *augustus080391* | AATGGGCGTCGGTGTCGTAC | GCGGCGTTGGTGTTGTATTTGG |
| *augustus090625* | GAATTAGTATTGGAAGGATGTGATGGG | TAAGCATGGGCGGCAACC |
| *augustus090626* | GAGCTATCATTACCTCCCTTCGCA | GCACTCATAACCTACCTCTTCCCTCG |
| *augustus037976* | CGTTGCTTCGACGGTGACGAGGAA | TCGGAGAGGTACAGTGGCACAGAC |
| *augustus101923* | TTGAGTCGTCTTGCTCTGGTGGGA | TCACTTCCTCCAAACTCGGCTCCT |
| *augustus016326* | AGGTGGACATGGTGGTAGCCCAAA | CCTGTTTCCGAGCAAACGCACACT |
| *augustus059604* | CACCGCAGCAACCTCTTCGTTCCA | ACGAGCGAGCATGATGAGGCAGAG |
| *augustus066272* | ACCGAGGTGTCAGGTGGAGGAACA | AAGCAATCGCAGCAACGTCGTGAG |
| *augustus068047* | AGGTCTTGGATGCCTGGTCCGATC | TGCTTCCCTTCTTGCCCTCTCCTC |
| *augustus074477* | GGACGGCAAGTGGATGGCTGTCAA | TCGGTGAGTTCCTTCGGTGGGCTA |
| *augustus095294* | ACATCAGCTTGGATTCGGGTCGGA | GTGGTGGTGGTGGTGGTTGGGAAA |
| CsAGD6-Hap-C1 | TCACATCAGCTTCGTCCGAT | TCGGTCTCTTTTGCGATGCC |
