# Supplementary figures and images for: Genome-wide association study and functional validation of CsAGD6 conferring drought tolerance in tea plant
Source: Hortic Res. 2025 Nov 21;13(3):uhaf320. doi: 10.1093/hr/uhaf320 (PMC12981331; doi:10.1093/hr/uhaf320)

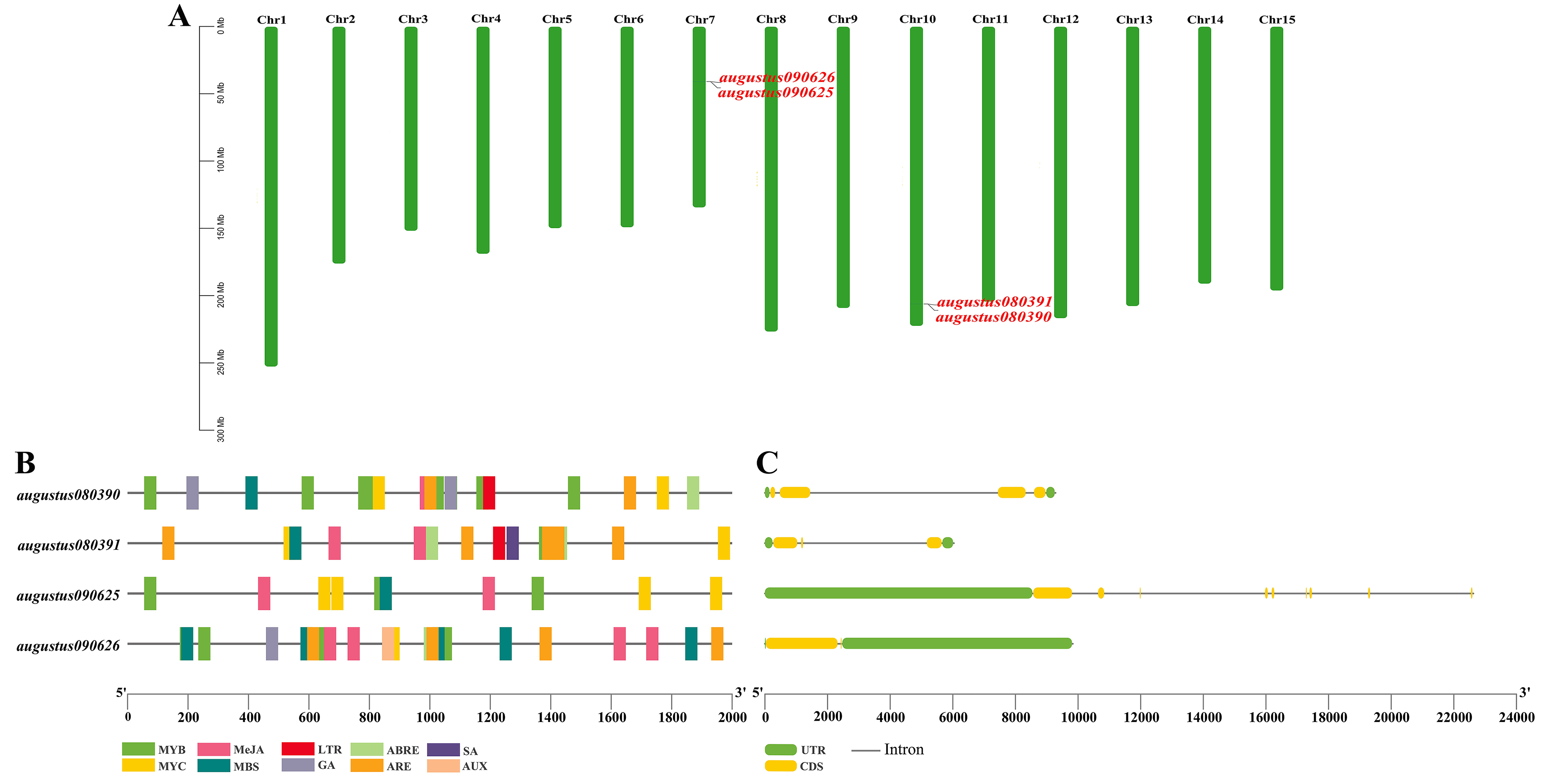

Supplement: Web_Material_uhaf320 [file web_material_uhaf320.zip › Fig.S1.tif]

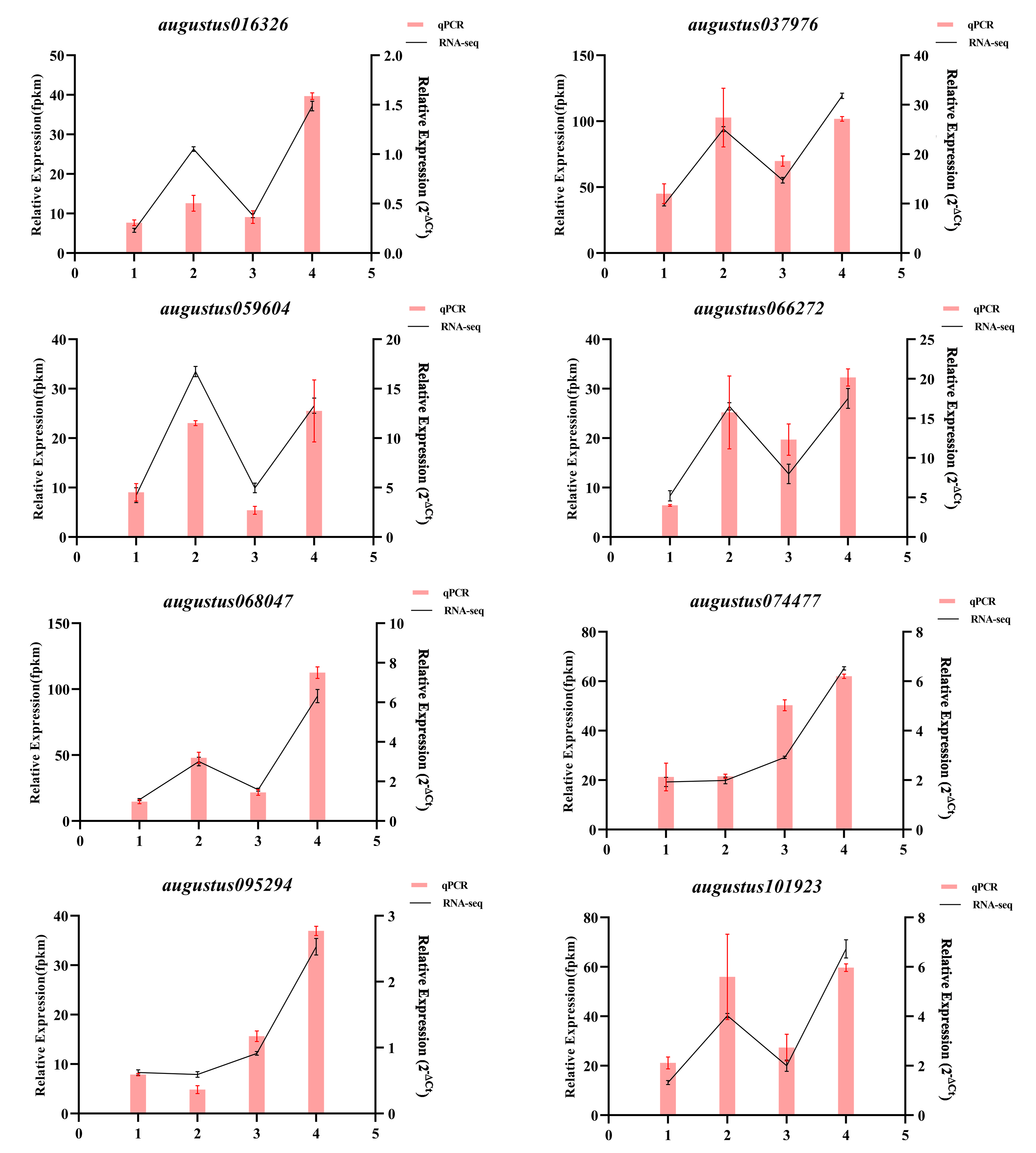

Supplement: Web_Material_uhaf320 [file web_material_uhaf320.zip › Fig.S2.tif]

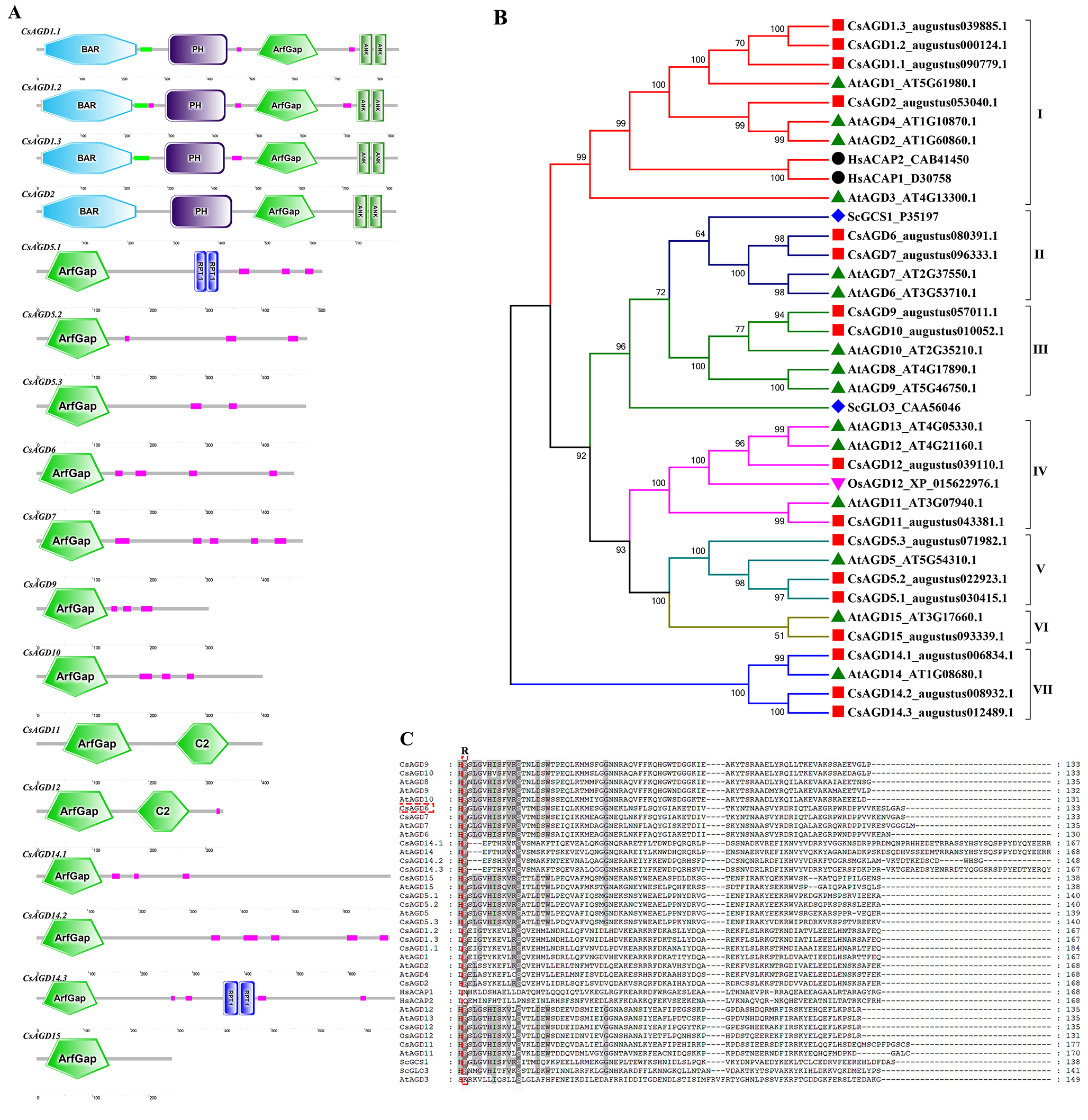

Supplement: Web_Material_uhaf320 [file web_material_uhaf320.zip › Fig.S3.tif]

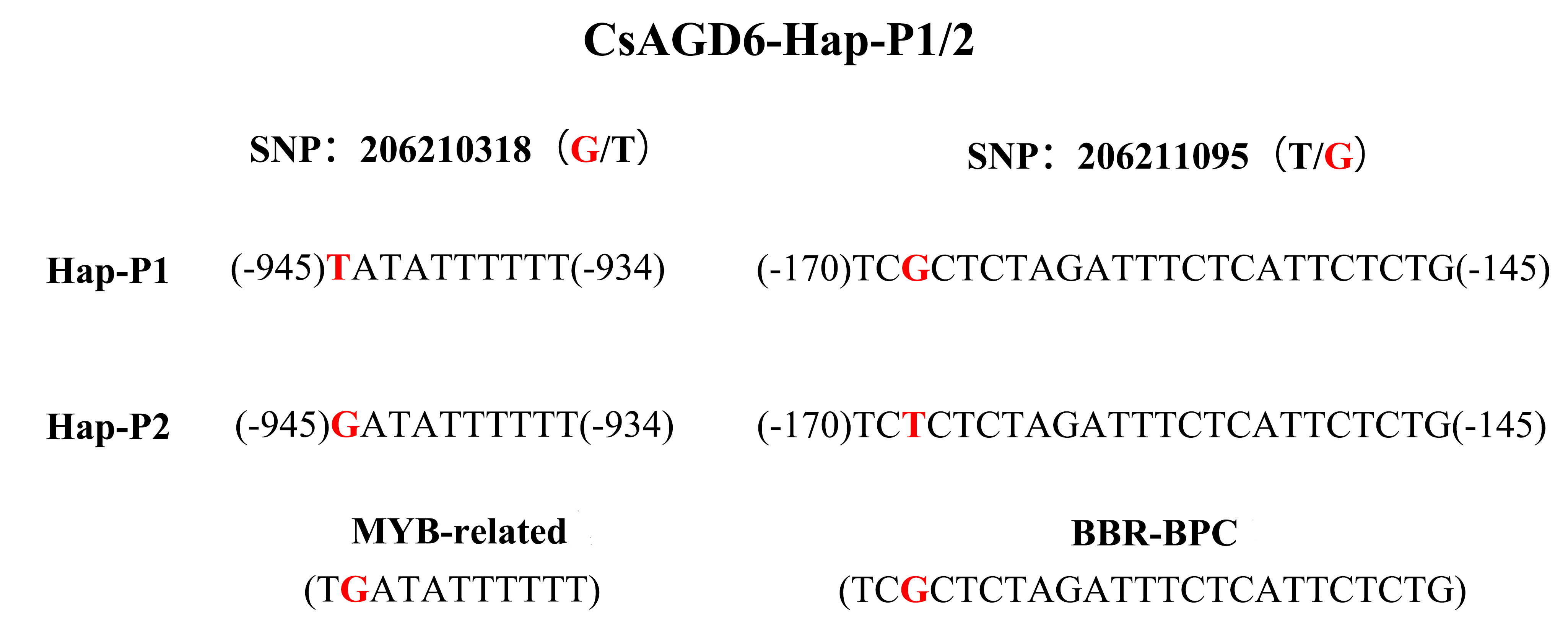

Supplement: Web_Material_uhaf320 [file web_material_uhaf320.zip › Fig.S4.tif]

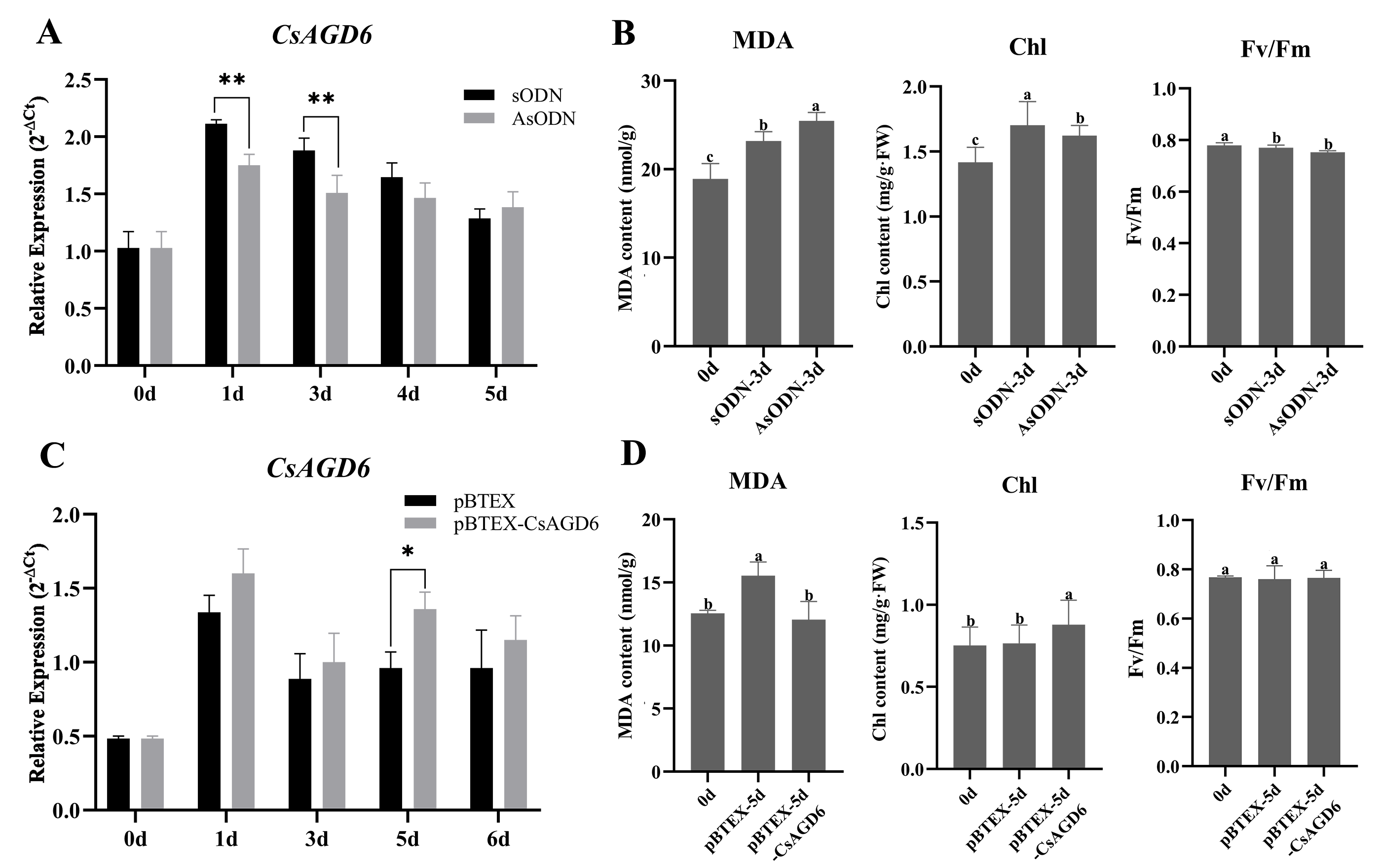

Supplement: Web_Material_uhaf320 [file web_material_uhaf320.zip › Fig.S5.tif]
